# PRMT6 Epigenetically Drives Metabolic Switch from Fatty Acid Oxidation toward Glycolysis and Promotes Osteoclast Differentiation During Osteoporosis

**DOI:** 10.1002/advs.202403177

**Published:** 2024-08-09

**Authors:** Wenxiang Chu, Weilin Peng, Yingying Lu, Yishan Liu, Qisheng Li, Haibin Wang, Liang Wang, Bangke Zhang, Zhixiao Liu, Lin Han, Hongdao Ma, Haisong Yang, Chaofeng Han, Xuhua Lu

**Affiliations:** ^1^ Department of Orthopaedic Surgery Changzheng Hospital Naval Medical University Shanghai 200003 China; ^2^ Obstetrics and Gynecology Hospital Fudan University Shanghai 200011 China; ^3^ Histology and Embryology Department and Shanghai Key Laboratory of Cell Engineering Naval Medical University Shanghai 200433 China; ^4^ Department of Orthopaedics Third Affiliated Hospital of Naval Medical University Shanghai 201805 China; ^5^ National Key Laboratory of Immunity and Inflammation, Institute of Immunology Naval Medical University Shanghai 200433 China

**Keywords:** fatty acids oxidation, glycolysis, metabolic reprogramming, osteoclastogenesis, PRMT6

## Abstract

Epigenetic regulation of metabolism profoundly influences cell fate commitment. During osteoclast differentiation, the activation of RANK signaling is accompanied by metabolic reprogramming, but the epigenetic mechanisms by which RANK signaling induces this reprogramming remain elusive. By transcriptional sequence and ATAC analysis, this study identifies that activation of RANK signaling upregulates PRMT6 by epigenetic modification, triggering a metabolic switching from fatty acids oxidation toward glycolysis. Conversely, *Prmt6* deficiency reverses this shift, markedly reducing HIF‐1α‐mediated glycolysis and enhancing fatty acid oxidation. Consequently, PRMT6 deficiency or inhibitor impedes osteoclast differentiation and alleviates bone loss in ovariectomized (OVX) mice. At the molecular level, *Prmt6* deficiency reduces asymmetric dimethylation of H3R2 at the promoters of genes including *Ppard*, *Acox3*, and *Cpt1a*, enhancing genomic accessibility for fatty acid oxidation. PRMT6 thus emerges as a metabolic checkpoint, mediating metabolic switch from fatty acid oxidation to glycolysis, thereby supporting osteoclastogenesis. Unveiling PRMT6's critical role in epigenetically orchestrating metabolic shifts in osteoclastogenesis offers a promising target for anti‐resorptive therapy.

## Graphical abstract

RANKL‐induced PRMT6 catalyzes asymmetric dimethylation of H3R2 at key FAO gene promoters (Ppard, Acox3, and Cpt1a), inhibiting FAO. This alleviates FAO's suppressive effect on glycolysis, synergizing with PRMT6’s promotion of HIF‐α‐dependent glycolysis, thus mediating the metabolic shift from FAO to glycolysis and promoting osteoclastogenesis initiation. RANKL: the receptor activator of nuclear factor kappa B ligand. FAO: fatty acids oxidation.

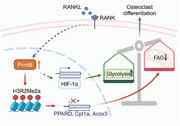

## 1 Introduction

Excessive osteoclastogenesis is a pivotal and common culprit in the progression of diseases such as osteoporosis, periodontitis, rheumatoid arthritis, and metastatic cancers.^[^ ^1^, ^2^, ^3^ ^]^ Specifically, in postmenopausal osteoporosis, estrogen deficiency lifts its suppression of osteoclast differentiation and elevates levels of the receptor activator of nuclear factor kappa B ligand (RANKL), resulting in excessive osteoclast activity and increased bone resorption.^[^ ^4^ ^]^ Given that anti‐resorptive therapies are the principal treatment approach for these conditions, a thorough investigation into the mechanisms underlying osteoclastogenesis is necessary to unveil new therapeutic targets.

Osteoclasts, bone tissue‐specific multinucleated cells derived from bone marrow monocytes/macrophages (BMMs), require two essential cytokines for differentiation: macrophage colony‐stimulating factor (M‐CSF) and RANKL.^[^ ^5^ ^]^ The activation of RANK by its ligand triggers a cascade of downstream signal transduction pathways, including NF‐κΒ, JNK, p38, ERK, and Src, which converge to initiate differentiation and activation.^[^ ^5^ ^]^ Meanwhile, metabolic reprogramming occurs in response to RANKL stimulation.^[^ ^6^, ^7^ ^]^ Activation of RANK signaling in BMMs increases glycolysis, and the genetic deletion of *Ldha* or *Glut1* impairs osteoclast differentiation.^[^ ^8^, ^9^, ^10^ ^]^ Moreover, increased aerobic glycolysis and glycolysis‐derived lactate production have been reported to be positively related to osteoclast‐mediated bone resorption.^[^ ^11^ ^]^ These findings indicate a close linkage between RANK signaling and metabolic reprogramming in osteoclastogenesis, yet the precise mechanisms of this connection remain to be elucidated.

Glucose and fatty acids, as primary energy substrates in biological activities, are increasingly recognized for their critical role in their metabolic switching that influencing cellular functions and disease progression.^[^ ^12^, ^13^, ^14^, ^15^ ^]^ A recent study discovered that reversing the metabolic switch from glycolysis to fatty acid oxidation (FAO) in cardiomyocytes enables heart regeneration in adult mice.^[^ ^16^ ^]^ Another study indicated that inhibition of PKM2 impairs EZH2 recruitment to SLC16A9, depressing its expression to boost intracellular carnitine influx, thereby reprogramming triple‐negative breast cancer cells into a FAO‐dependent state for survival in glycolysis‐deficient environments.^[^ ^17^ ^]^ However, little is known about how the metabolic priority between glycolysis and FAO is determined during osteoclastogenesis.

Protein arginine methyltransferase 6 (PRMT6), a type 1 PRMT, asymmetrically dimethylates the arginine residues of certain proteins, including histones, transcriptional factors, and coregulators.^[^ ^18^, ^19^ ^]^ Epigenetic regulation plays a fundamental role in mediating various cellular processes, with histone modification being a crucial form of such regulation. In the context of histone modification, the catalytic sites of PRMT6 are found at arginine 2 (H3R2me2a), arginine 17 (H3R17me2a), and arginine 42 (H3R42me2a) on histone H3, as well as at arginine 26 (H2AR26me2a) on histone H2A.^[^ ^19^ ^]^ H3R2me2a is known as a repressive histone modification that reduces chromatin accessibility through mutual exclusion with H3K4me3.^[^ ^20^ ^]^ To date, research findings have revealed the significant role of PRMT6 in tumorigenesis, viral diseases, neurodegeneration,^[^ ^21^, ^22^, ^23^, ^24^, ^25^ ^]^ as well as many physiological functions such as mitosis,^[^ ^26^, ^27^ ^]^ DNA repair,^[^ ^28^ ^]^ senescence,^[^ ^29^ ^]^ development and differentiation,^[^ ^30^, ^31^ ^]^ among others. However, the roles and mechanisms by which PRMT6 regulates osteoclastogenesis and metabolic reprogramming remain elusive.

In this study, utilizing multi‐omics analysis, we identified PRMT6 as a novel metabolic checkpoint that mediates the shift from fatty acid oxidation to glycolysis, converting RANK signaling into metabolic switching during osteoclast differentiation. *Prmt6* deficiency reverses this shift, markedly reducing HIF‐1α‐mediated glycolysis while enhancing fatty acid oxidation. This reversal, induced either by PRMT6 deficiency or pharmacological inhibition, significantly impedes osteoclast differentiation and mitigates bone loss in ovariectomized (OVX) mice. At the molecular level, *Prmt6* deficiency leads to a reduction in asymmetric dimethylation of H3R2 at the promoters of key gene loci implicated in fatty acid oxidation, including *Ppard*, *Acox3*, and *Cpt1a*. This reduction removes the repressive effects of the H3R2me2a modification on those genes, thus enhancing their genomic accessibility and promoting fatty acid oxidation pathways. Our findings highlight the epigenetic role of PRMT6 in metabolic reprogramming during osteoclastogenesis and suggest it as a potential therapeutic target for anti‐resorptive therapy.

## 2 Results

### 2.1 Omics Analysis Reveals an Upregulation of PRMT6 in Response to RANK Signaling Activation

**Figure 1:**
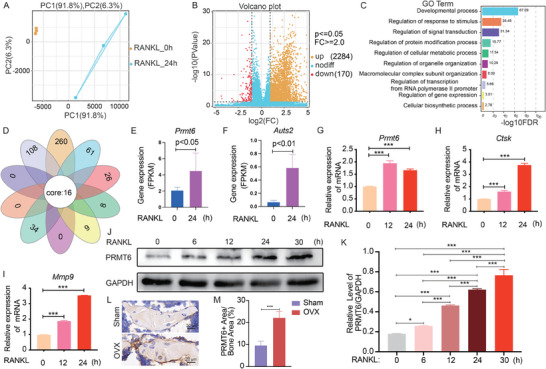
Omics analysis reveals an upregulation of PRMT6 in response to RANK signaling activation. A) An osteoclast differentiation model was constructed through inducing mouse bone marrow monocytes/macrophages (BMMs) with RANKL, followed by RNA‐sequencing analysis. Principal Component Analysis (PCA) demonstrated distinct segregation of samples 24 h post‐RANKL induction from those in pre‐induction states, underscoring significant gene expression alterations driven by RANK signaling activation. B) Volcano plot illustrating differentially expressed genes (DEGs) in BMMs before and after 24 h of RANKL induction (FC ≥ 2, p ≤ 0.05). C) Gene Ontology (GO) enrichment analysis reveals significantly enriched biological processes, with a focus on GO terms related to metabolic regulation and molecular mechanisms, which are highlighted and presented. D) The Venn diagram showcasing the intersection among the ten biological processes of interest, aimed at identifying candidate genes common to both metabolic regulation and molecular mechanism control. E,F) Quantification of gene expression for *Prmt6* (E) and *Auts2* (F), highlighting two genes previously unidentified in osteoclastogenesis, as derived from the core of the Venn diagram analysis. G–I) In vitro gene expression analysis of *Prmt6* (G), *Ctsk* (H), and *Mmp9* (I) in RANKL‐induced BMMs as assessed by quantitative PCR. J) Representative Western blot showing the expression levels of PRMT6 during the differentiation of BMMs into osteoclasts, induced by RANKL. K) Quantitative analysis of PRMT6 protein levels at different time points during osteoclastogenesis. L) Immunohistochemical staining showing the localization and increased expression of PRMT6 in bone sections from ovariectomized (OVX) mice compared to sham‐operated controls. M) Quantitative analysis of PRMT6 expression levels in bone tissue, derived from immunohistochemical staining intensities.

To identify potential mediators of metabolic reprogramming induced by RANK signaling, BMMs were exposed to RANKL, followed by RNA sequencing at 24 h post‐induction. Principal component analysis revealed distinct clustering of samples before and after RANKL stimulation, indicating significant transcriptional changes (**Figure** **1**A). Analysis identified 2454 differently expressed genes (DEGs) (FC ≥ 2, p < 0.05), with 2284 genes upregulated and 170 genes downregulated (Figure 1B). Subsequent Gene Ontology (GO) enrichment analysis on these DEGs highlighted significant enrichment in various biological processes. In order to hone in on genes relevant to metabolic reprogramming, we particularly focused on the GO term “Regulation of cellular metabolic process” (Figure 1C). Alongside, we also examined additional significantly enriched GO terms related to our differentiation model and molecular mechanism regulation, such as “Developmental process”, “Regulation of response to stimulus”, “Regulation of signal transduction”, “Regulation of protein modification process”, among others (Figure 1C). Employing Venn diagram analysis (Figure 1D) across these ten pertinent GO terms, we pinpointed candidate genes implicated in both metabolic reprogramming and the regulation of molecular mechanisms during osteoclast differentiation. This approach identified sixteen overlapping DEGs, prominently featuring *Prmt6* (Figure 1E) and *Auts2* (Figure 1F) as significantly upregulated, while the remaining 14 DEGs have been previously recognized in the context of osteoclastogenesis (**Table** **1**). Given *Prmt6*’s higher expression relative to *Auts2*, we focused on *Prmt6* as a key candidate for mediating RANK signaling‐induced metabolic reprogramming. We subsequently used BMMs stimulated with RANKL for 12 h to perform ATAC‐seq. Analysis of the ATAC‐seq data also revealed increased chromatin accessibility at the *Prmt6* locus and the osteoclast marker *Mmp9* following RANKL induction (Figure S1, Supporting Information).

**Table 1:** Sixteen overlapping DEGs in the Venn diagram from Figure 1D.

Gene	Log_2_(FC)	*p‐*value
*Gfi1* ^*^	3.60	<0.05
*Cav1* ^*^	3.54	<0.01
*Auts2* ^#^	3.12	<0.01
*Cdkn2a* ^*^	3.07	<0.01
*Tgfb2* ^*^	2.83	<0.01
*Snai2* ^*^	2.81	<0.01
*Twist1* ^*^	2.68	<0.01
*Lif* ^*^	2.68	<0.01
*Brca1* ^*^	2.61	<0.01
*Prkd1* ^*^	2.59	<0.01
*Id1* ^*^	2.57	<0.01
*Gata2* ^*^	2.21	<0.01
*Cx3cl1* ^*^	1.78	<0.01
*Smad3* ^*^	1.63	<0.01
*Prmt6* ^#^	1.11	<0.05
*Trp53* ^*^	1.04	<0.01

Note: Asterisks indicate genes previously reported in association with osteoclasts, while pound signs denote genes not yet reported in osteoclast research.

We further validated the upregulation of PRMT6 at both the mRNA (Figure 1G–I) and protein levels (Figure 1J,K) in RANKL‐induced BMMs. Given the overactivation of RANK signaling in OVX conditions, we compared PRMT6 expression in bone tissues from OVX and Sham‐operated groups. Our findings revealed that PRMT6 was significantly elevated in OVX bone tissue (Figure 1L,M).

Collectively, these results identified PRMT6 as a potential metabolic regulator, upregulated in response to RANK signaling activation.

### 2.2 *Prmt6* Deficiency Inhibits RANKL‐Induced Osteoclastogenesis In Vitro

**Figure 2:**
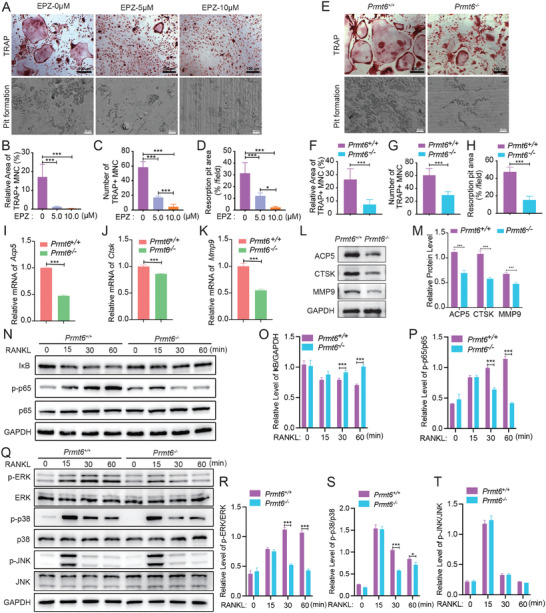
PRMT6 deficiency inhibits RANKL‐induced osteoclast differentiation and activation in vitro. A) The PRMT6 inhibitor (EPZ020411) suppressed TRAP+ multinucleated cell (TRAP+/MNC) formation and osteoclast‐mediated pit formation in a dose‐dependent manner. B,C) Quantitative analysis of relative area (B) or number (C) of TRAP+/MNC under the administration of 0, 5.0, and 10 µm PRMT6 inhibitor. D) Quantitative analysis of osteoclast‐mediated pit formation area under the administration of PRMT6 inhibitor. E) *Prmt6* knockout inhibited TRAP+ osteoclast formation and osteoclast‐mediated pit formation. F,G) Quantitative analysis of relative area (F) or number (G) of TRAP+/MNC between RANKL‐induced *Prmt6^+/+^* and *Prmt6^−/−^* BMMs. H) Quantitative analysis of the area of pit formation on bovine bone slices, cultured with RANKL‐induced *Prmt6^+/+^* or *Prmt6^−/−^* BMMs. I–K) Analysis of osteoclast marker gene expression levels in RANKL‐induced BMMs, showing a significant reduction in *Acp5* (I), *Ctsk* (J), and *Mmp9* (K) following *Prmt6* knockout. L) *Prmt6* knockout resulted in decreased protein expression levels of ACP5, CTSK, and MMP9 in BMMs at 4 days post‐RANKL induction. M) Quantitative analysis of the protein expression levels of ACP5, CTSK, and MMP9 in *Prmt6^+/+^* and *Prmt6^−/−^* BMMs at 4 days post‐RANKL induction. N) Representative Western blot analysis showing the inhibition of the NF‐κB signaling pathway in BMMs induced by RANKL in the absence of *Prmt6*. O) Quantitative analysis of IkB protein levels in BMMs following RANKL stimulation at 30 and 60 min. *Prmt6* knockout BMMs exhibit significantly higher IkB levels compared to wild‐type controls, indicating inhibited NF‐κB pathway activation. P) The analysis reveals significantly lower levels of phosphorylated p65 relative to total p65 (p‐p65/p65) in *Prmt6*‐deficient BMMs at both 30 and 60 min post‐RANKL induction. Q) Western blot analysis demonstrating that *Prmt6* knockout significantly reduces the phosphorylation of ERK (p‐ERK) and p38 (p‐p38) in BMMs after RANKL induction, whereas the phosphorylation levels of JNK (p‐JNK) remain unaffected. R) Quantitative analysis shows significantly lower p‐ERK levels in *Prmt6* knockout BMMs at 30 and 60 min following RANKL stimulation, compared to wild‐type cells. S) Similarly, Prmt6 deficiency results in markedly reduced p‐p38 levels at both time points under RANKL induction. T) The levels of p‐JNK in *Prmt6*‐deficient BMMs do not show significant differences compared to wild‐type cells.

To investigate PRMT6's contribution to RANKL‐induced osteoclastogenesis, we employed the selective PRMT6 inhibitor, EPZ020411, throughout the entire differentiation process. Notably, PRMT6 inhibition led to a dose‐dependent reduction in the formation of multinucleated, TRAP‐positive cells (TRAP+/MNCs) (**Figure** **2**A–C; Figure S2A, Supporting Information) and reduced bone erosion caused by osteoclasts (Figure 2A,D). A comparative analysis between *Prmt6^+/+^* and *Prmt6^−/−^* BMMs revealed that while *Prmt6^+/+^* BMMs developed into matured osteoclasts capable of significant bone resorption, *Prmt6^−/−^* cells showed markedly fewer and smaller TRAP+/MNCs (Figure 2E–G), and reduced bone resorption activity (Figure 2E,H). Additionally, *Prmt6* deficiency was associated with decreased mRNA levels of osteoclast markers ACP5, CTSK, and MMP9 (Figure 2I–K), and lower protein levels at 4 days of differentiation (Figure 2L,M). Considering the established roles of NF‐κB and MAPK pathways in osteoclastogenesis, we assessed their activation in the context of *Prmt6* deficiency. In *Prmt6^+/+^* cells, RANKL stimulation led to rapid phosphorylation of p65, ERK, JNK, and p38. Contrastingly, *Prmt6^−/−^* cells showed reduced activation of p65, ERK, and p38, while JNK phosphorylation was unaffected (Figure 2N–T). Furthermore, we explored PRMT6’*s* effect on the osteogenic differentiation of bone marrow mesenchymal stem cells (BMSCs), finding that neither inhibition by EPZ020411 nor deficiency significantly altered ALP expression or bone mineral formation in osteogenically induced BMSCs (Figure S2B–H, Supporting Information). These results collectively demonstrate that PRMT6 promotes RANKL‐induced osteoclastogensis in vitro, without appreciably influencing osteogenic differentiation.

### 2.3 *Prmt6* Deficiency Attenuates OVX‐Induced Bone Loss

**Figure 3:**
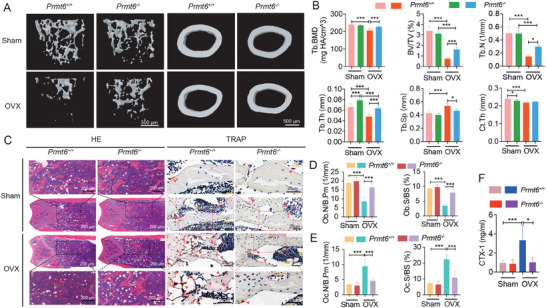
*Prmt6* deficiency attenuates OVX‐induced bone loss and osteoclastogenesis in vivo. A) Micro‐CT images from *Prmt6^−/−^* mice and their wild‐type littermates demonstrate that *Prmt6* deficiency significantly mitigates OVX‐induced trabecular bone loss with minimal impact on cortical bone. B) Quantitative analyses of trabecular and cortical bone parameters in femurs using micro‐CT, including bone mineral density (BMD), bone volume fraction (BV/TV), trabecular number (Tb.N), trabecular thickness (Tb.Th), trabecular separation (Tb.Sp), and cortical thickness (Ct. Th). C) Hematoxylin and eosin (HE) and tartrate‐resistant acid phosphatase (TRAP) staining of decalcified sections of distal femurs from *Prmt6^−/−^* and their wild‐type littermates. HE staining (left panels) reveals reduced OVX‐induced trabecular bone loss in *Prmt6^−/−^* mice while TRAP staining (right panels) indicates fewer TRAP+ osteoclasts, marked by red arrows. D) Histomorphometric analyses of distal femurs, showing the number of osteoblasts per bone perimeter (Ob.N/B.Pm) and the osteoblast surface area per bone surface area (Ob.S/BS). E) Histomorphometric analyses of distal femurs, showing the number of osteoclasts per bone perimeter (Oc.N/B.Pm) and the osteoclast surface area per bone surface area (Oc.S/BS). F) Measurement of serum levels of C‐terminal telopeptide type 1 collagen (CTX‐1) in *Prmt6^+/+^* and *Prmt6^−/−^* mice, with and without OVX treatment.

Given the increased osteoclastogenesis driven by elevated RANKL levels under OVX conditions, we examined the role of PRMT6 in OVX‐induced bone loss. Micro‐CT analyses in Sham‐operated mice revealed no significant differences between *Prmt6^−/−^* and *Prmt6^+/+^* groups in trabecular bone mineral density (Tb.BMD), bone volume fraction (BV/TV), trabecular number (Tb.N), and trabecular separation (Tb.Sp) (**Figure** **3**A,B). Interestingly, *Prmt6^−/−^* mice exhibited a slightly higher trabecular thickness (Tb.Th), while their cortical thickness (Ct.Th) was marginally reduced compared to *Prmt6^+/+^* mice (Figure 3B). Five weeks post‐OVX, *Prmt6^−/−^* mice showed significantly improved bone microarchitecture compared to *Prmt6^+/+^* mice. This improvement encompassed enhanced Tb.BMD, BV/TV, Tb.N, Tb.Th and Tb.Sp (Figure 3A,B).

Histomorphometric analysis further corroborated these findings. In the Sham condition, there were no significant differences in osteoblast numbers per bone perimeter (Ob.N/B.Pm) or osteoblast surface per bone surface (Ob.S/BS) between *Prmt6^−/−^* and *Prmt6^+/+^* mice (Figure 3C,D). Similarly, osteoclast numbers per bone perimeter (Oc.N/B.Pm) and osteoclast surface per bone surface (Oc.S/BS) were comparable between the groups (Figure 3C,E).

However, following OVX treatment, *Prmt6^−/−^* mice demonstrated a significant increase in both Ob.N/B.Pm and Ob.S/BS (Figure 3C,D), and a significant decrease in both Oc.N/B.Pm and Oc.S/BS in trabecular femoral bone sections compared to their *Prmt6^+/+^* counterparts (Figure 3C,E). Additionally, serum levels of C‐terminal telopeptide of type 1 collagen (CTX‐1) were markedly lower in OVX‐treated *Prmt6^−/−^* mice, indicating reduced bone resorption and osteoclast activity (Figure 3F).

Collectively, these results underscore PRMT6's pivotal role in promoting osteoclastogenesis and accelerating bone loss under OVX conditions.

### 2.4 *Prmt6* Deficiency Reduces the HIF‐1 Signaling Pathway in RANKL‐Induced BMMs

To elucidate the mechanism by which PRMT6 influences RANKL‐induced osteoclastogenesis, we performed a comprehensive analysis of BMMs from *Prmt6^−/−^* and *Prmt6^+/+^* mice, both untreated and treated with RANKL, utilizing ATAC‐seq, RNA‐seq, and quantitative LC/LM proteomics.

**Figure 4:**
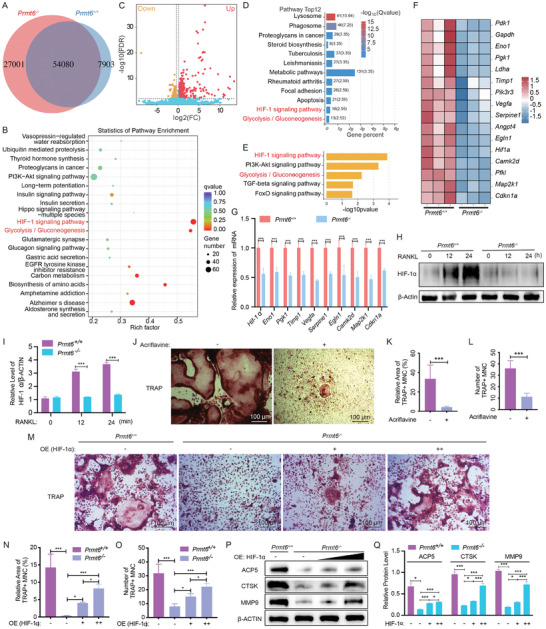
*Prmt6* deficiency reduces the HIF‐1 signaling pathway in RANKL‐induced BMMs. A) ATAC‐seq showed the number of accessible chromatin regions (peaks) in *Prmt6^−/−^* compared to *Prmt6^+/+^* BMMs post‐12‐h RANKL stimulation, indicating altered chromatin accessibility. B) KEGG enrichment analysis for genes associated with differential peaks between *Prmt6^−/−^* and *Prmt6^+/+^* BMMs post‐12‐h RANKL stimulation. A log_2_|Fold change| > 1 between *Prmt6^−/−^* and *Prmt6^+/+^* RPM values indicates the differential peaks, with proximal genes considered associated. C) A Volcano plot illustrating DEGs (Fold change >= 1.2, FDR <= 0.01) between *Prmt6^−/−^* and *Prmt6^+/+^* cells upon 24‐h RANKL stimulation, derived from RNA‐seq data. D) The Top 12 significantly enriched KEGG pathways based on the DEGs between *Prmt6^−/−^* and *Prmt6^+/+^* cells, as identified from RNA‐seq data, reveal key pathways impacted by *Prmt6* deficiency. E) RNA‐seq data analysis shows significantly downregulated pathways in the KEGG signaling transduction category and the Glycolysis/Gluconeogenesis pathway in *Prmt6^−/−^* BMMs compared to *Prmt6^+/+^* counterparts. F) A heatmap displays genes within the HIF‐1 signaling pathway, contrasting *Prmt6^−/−^* and *Prmt6^+/+^* BMMs after 24 h of RANKL stimulation, underscoring the downregulation of these HIF‐1 signaling genes. G) qPCR analysis validated the decreased gene expression in *Prmt6^−/−^* cells relative to *Prmt6^+/+^* cells following 24 h of RANKL induction. H) Western blot analysis demonstrating that *Prmt6* knockout leads to a decreased protein expression level of HIF‐1α during in vitro osteoclastogenesis induced by RANKL. I) Quantitative analysis confirming that *Prmt6* knockout results in significantly decreased protein expression levels of HIF‐1α in BMMs during RANKL‐induced osteoclastogenesis. J) The inhibition of HIF‐1α using Acriflavine significantly reduces the formation of TRAP+ osteoclasts, demonstrating the crucial role of HIF‐1α in osteoclastogenesis. K,L) Quantitative analysis demonstrated the inhibitory effect of Acriflavine on TRAP+ osteoclast formation. M) TRAP staining shows that HIF‐1α overexpression partially rescues the formation of TRAP+ osteoclasts in *Prmt6*‐deficient BMMs. N,O) Quantitative analysis indicates that HIF‐1α overexpression partially rescues the relative area (N) and number (O) of TRAP+ osteoclasts in *Prmt6*‐deficient BMMs. P) Western blot analysis shows that HIF‐1α overexpression partially rescues the protein levels of osteoclast markers ACP5, CTSK, and MMP9 in *Prmt6*‐deficient BMMs. Q) Quantitative analysis demonstrates that HIF‐1α overexpression partially rescues the protein levels of osteoclast markers ACP5, CTSK, and MMP9 in *Prmt6*‐deficient BMMs.

ATAC‐seq analysis, conducted after 12 h of RANKL exposure, revealed decreased chromatin accessibility at the osteoclast marker gene Mmp9 in *Prmt6^−/−^* cells compared to *Prmt6^+/+^* cells (Figure S1, Supporting Information). Across the genome, ATAC‐seq identified 54080 accessible regions (peaks) shared between the two cell types, with 27001 peaks unique to *Prmt6^−/−^* cells and 7903 unique to *Prmt6^+/+^* cells (**Figure** **4**A). Notably, differential peak analysis between RANKL‐stimulated *Prmt6^−/−^* and *Prmt6^+/+^* cells showed significant enrichment of the HIF‐1 signaling and Glycolysis/Gluconeogenesis KEGG pathways, highlighted by the highest enrichment factors (Figure 4B).

Subsequent RNA‐seq analysis of DEGs post 24‐h RANKL stimulation indicated significant enrichment in these pathways among the top 12 identified pathways (Figure 4C,D). KEGG enrichment analysis on downregulated genes in *Prmt6^−/−^* BMMs highlighted the HIF‐1 signaling pathway as the most affected within the signal transduction category, with Glycolysis/Gluconeogenesis also significantly enriched (Figure 4E). Key genes in the HIF‐1 pathway exhibited reduced expression levels in *Prmt6^−/−^* cells (Figure 4F), as corroborated by qPCR, which confirmed the downregulation of Hif‐1α and other pathway components (Figure 4G).

Given the notable decrease in Hif‐1α transcription in *Prmt6^−/−^* cells and its central role in HIF‐1 signaling, we further assessed HIF‐1α protein expression during RANKL‐induced osteoclastogenesis. *Prmt6^+/+^* cells displayed elevated levels of HIF‐1α, whereas *Prmt6^−/−^* cells showed sustained inactivation of HIF‐1α (Figure 4H,I). Pre‐treatment of BMMs with the HIF‐1α inhibitor Acriflavine resulted in significantly reduced osteoclast formation (Figure 4J–L). Similarly, the application of α‐KG, which enhances prolyl hydroxylase domain (PHD) enzyme activity leading to HIF‐1α degradation,^[^ ^32^ ^]^ markedly impaired osteoclastogenesis in vitro (Figure S3A–C, Supporting Information). Moreover, overexpression of HIF‐1α partially rescued the formation of TRAP‐positive osteoclasts (Figure 4M–O) and partially restored levels of osteoclast markers ACP5, CTSK, and MMP9 in RANKL‐induced *Prmt6^−/−^* BMMs (Figure 4P,Q).

These results highlight PRMT6's crucial involvement in modulating the HIF‐1 signaling pathway, providing insights into its role in the molecular mechanisms governing osteoclast formation and activity.

### 2.5 *Prmt6* Deficiency Shifts Metabolism from Glycolysis toward Fatty Acid Oxidation in RANKL‐Induced BMMs

**Figure 5:**
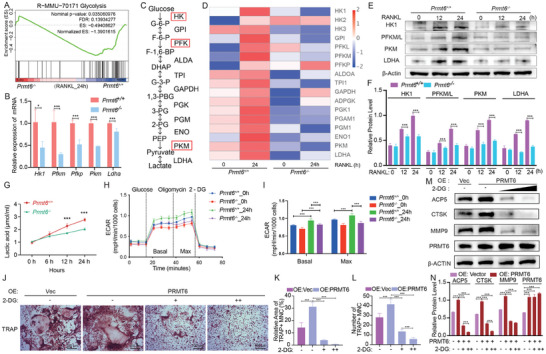
*Prmt6* deficiency inhibits glycolysis in RANKL‐induced BMMs. A) GSEA analysis revealed significantly inhibited glycolysis in Prmt6*^−^* ^/^ *^−^* BMMs compared to *Prmt6^+/+^* BMMs following 24‐h RANKL stimulation. B) qPCR confirmed that *Prmt6* deficiency significantly reduced the expression of glycolysis‐related genes in BMMs after 24 h of RANKL induction. C) Schematic of the enzymes and metabolites involved in the glycolysis pathway. D) A heatmap displayed the proteomic profiling of glycolytic enzymes, illustrating significant upregulation in response to RANKL in wild‐type BMMs, an effect mitigated by *Prmt6* deficiency. E) Comparative analysis of glycolytic enzyme expression (HK1, PFKM/L, PKM, and LDHA) between *Prmt6^−/−^* and *Prmt6^+/+^* BMMs during RANKL stimulation, corroborating the findings from proteomic profiling. F) Quantitative analysis shows that *Prmt6* deficiency significantly inhibits RANKL‐induced expression of glycolytic enzymes (HK1, PFKM/L, PKM, and LDHA). G) Quantification of lactate secretion in cell culture supernatants from *Prmt6^−/−^* and *Prmt6^+/+^* BMMs under RANKL stimulation. H,I) Analysis of extracellular acidification rate (ECAR) revealed a decrease in *Prmt6^−/−^* BMMs compared to *Prmt6^+/+^* counterparts after 24‐h RANKL stimulation. J) Inhibition of glycolysis with 2‐DG significantly abolishes the enhancement of TRAP+ osteoclast formation induced by PRMT6 overexpression during RANKL stimulation. K,L) Quantitative analysis shows that 2‐DG inhibition of glycolysis significantly abolishes the relative area (K) and number (L) of TRAP+ osteoclasts induced by PRMT6 overexpression. M) Western blot analysis indicates that 2‐DG inhibition of glycolysis significantly abolishes the elevated protein levels of osteoclast markers ACP5, CTSK, and MMP9 induced by PRMT6 overexpression. N) Quantitative analysis demonstrates that 2‐DG inhibition of glycolysis significantly abolishes the elevated protein levels of osteoclast markers ACP5, CTSK, and MMP9 induced by PRMT6 overexpression.

Given HIF‐1α’s well‐established role in regulating glycolysis and the observed enrichment in the Glycolysis/Gluconeogenesis pathway, we explored how *Prmt6* deficiency influences glycolysis during osteoclastogenesis. Gene Set Enrichment Analysis (GSEA) of RNA‐seq data from RANKL‐stimulated cells revealed a significant reduction in glycolysis in *Prmt6^−/−^* samples (**Figure** **5**A). qPCR corroborated this finding, showing markedly decreased expression of essential glycolytic genes including *Hk1*, *Pfkm/p*, *Pkm*, and *Ldha* in *Prmt6^−/−^* cells (Figure 5B). Proteomic analysis further demonstrated an extensive upregulation of glycolytic enzymes in *Prmt6^+/+^* BMMs following RANKL stimulation, an effect substantially diminished in *Prmt6^−/−^* cells (Figure 5C,D). Western Blot analysis supported these observations, showing a significant inhibition in the upregulation of key glycolytic enzymes (HK1, PFKM/L, PKM, and LDHA) in *Prmt6^−/−^* cells at 12 and 24 h post‐RANKL induction (Figure 5E,F). Lactate production, a key glycolytic end‐product, was significantly lower in *Prmt6^−/−^* BMMs than in *Prmt6^+/+^* cells during osteoclastogenesis (Figure 5G). Evaluation of glycolytic activity through extracellular acidification rate (ECAR) measurements at 24 h post‐induction revealed a significant decrease in *Prmt6^−/−^* BMMs compared to *Prmt6^+/+^* counterparts (Figure 5H,I). Thus, *Prmt6* deficiency significantly inhibited RANKL‐activated glycolysis.

Inhibition of glycolysis with 2‐deoxyglucose (2‐DG) significantly blocked osteoclastogenesis (Figure S3D–F, Supporting Information). Moreover, the enhanced formation of TRAP+ osteoclasts due to PRMT6 overexpression was entirely abolished by 2‐DG‐mediated inhibition of glycolysis (Figure 5J–L). Similarly, the upregulation of osteoclast markers ACP5, CTSK, and MMP9 induced by PRMT6 overexpression was fully reversed by 2‐DG treatment (Figure 5M–N). These findings collectively suggest that the inhibition of glycolysis in the absence of PRMT6 is a crucial factor in the impaired osteoclastogenesis observed in *Prmt6*‐deficient cells.

**Figure 6:**
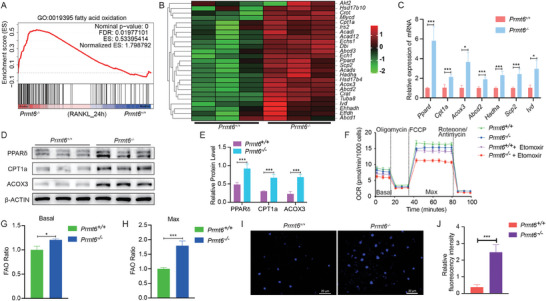
*Prmt6* deficiency increases fatty acid oxidation (FAO) in RANKL‐induced BMMs. A) GSEA revealed an augmentation in FAO in *Prmt6^−/−^* BMMs compared to *Prmt6^+/+^* BMMs post‐24‐h RANKL induction. B) Heatmap presenting upregulated genes involved in the FAO in *Prmt6^−/−^* BMMs post‐24‐h RANKL induction. C) RT‐qPCR analysis confirmed the upregulation of FAO‐related genes in *Prmt6^−/−^* BMMs relative to *Prmt6^+/+^* counterparts after 24‐h RANKL stimulation. D) Western blot analysis revealed that *Prmt6* deficiency significantly increased the levels of FAO‐related proteins PPARδ, CPT1a, and ACOX3 at 24 h post‐RANKL induction. E) Quantitative analysis showed that *Prmt6* deficiency significantly increased the levels of FAO‐related proteins PPARδ, CPT1a, and ACOX3 at 24 h post‐RANKL induction. F) OCR quantifying FAO in 24‐h RANKL‐induced *Prmt6^+/+^*, *Prmt6^−/−^*, *Prmt6^+/+^* + Etomoxir, and *Prmt6^−/−^* + Etomoxir BMMs. G,H) FAO ratio at Basal (G) and Max (H) OCR levels, calculated as [OCR (*Prmt6^−/−^*) − OCR (*Prmt6^−/−^* + Etomoxir)]/[OCR (*Prmt6^+/+^*) − OCR (*Prmt6^+/+^* + Etomoxir)]. I,J) Blue fluorescence imaging and intensity quantification following FAOBlue reagent application for 40 min on RANKL‐induced *Prmt6^+/+^* and *Prmt6^−/−^* BMMs at 24 h, showing an increased FAO activity in *Prmt6^−/−^* BMMs.

To investigate broader metabolic changes, we performed GSEA on RNA‐seq data from RANKL‐stimulated cells, focusing on major metabolic pathways affected by *Prmt6* deficiency. Interestingly, *Prmt6^−/−^* cells exhibited significant upregulation in fatty acid oxidation (FAO) pathways compared to *Prmt6^+/+^* cells (**Figure** **6**A). This upregulation was confirmed by increased expression of FAO‐related genes, including *Ppard*, *Cpt1a*, and *Acox3*, as validated by qPCR (Figure 6B,C). Additionally, protein levels of PPARδ, CPT1a, and ACOX3 were elevated in RANKL‐induced *Prmt6^−/−^* BMMs (Figure 6D,E). Functional assays further substantiated these findings. By measuring the Oxygen Consumption Rate (OCR) before and after Etomoxir treatment, we found that *Prmt6^−/−^* BMMs maintained significantly higher FAO levels compared to *Prmt6^+/+^* cells at 24 h post‐RANKL induction (Figure 6F–H). This elevated FAO activity in *Prmt6^−/−^* BMMs was also evidenced by higher FAOBlue fluorescence intensity following RANKL induction (Figure 6I,J). These results indicate that *Prmt6*‐deficient BMMs depend more heavily on FAO during early RANKL‐induced osteoclastogenesis compared to their wild‐type counterparts. In vivo, under the OVX context with excessive RANK activation, *Prmt6* deficiency significantly inhibited the increase in key glycolysis components HIF‐1a and LDHA in bone marrow cells (Figure S4A–D, Supporting Information), while also preventing the reduction in key FAO components PPARδ and CPT1a (Figure S4E–H, Supporting Information). This further suggests that PRMT6 has opposing effects on glycolysis and FAO, contributing to osteoclastogenesis and bone loss.

To further elucidate the metabolic shifts during normal osteoclast differentiation, we conducted GSEA to compare changes in glycolysis and FAO at 24 h post‐RANKL induction in *Prmt6^+/+^* cells. Our analysis revealed a significant upregulation in glycolysis (Figure S5A, Supporting Information) alongside a concurrent suppression of FAO (Figure S5B, Supporting Information). This was supported by a substantial increase in the expression of glycolytic genes such as *Pfkl*, *Pkm*, and *Ldha* during osteoclastogenesis (Figure S5C, Supporting Information), as well as elevated protein levels of these enzymes (Figure S5D,E, Supporting Information). Conversely, RNA‐seq data indicated a broad downregulation of FAO‐related genes 24 h post‐RANKL induction (Figure S5F, Supporting Information). qPCR confirmed a significant reduction in the expression of *Ppard*, *Cpt1a*, *Acadl, Acox1, Hadha* and *Acox3* (Figure S5G, Supporting Information), and protein levels of these FAO components also decreased markedly following 24 h of RANKL treatment (Figure S5H,I, Supporting Information). Furthermore, FAO activity, as measured by OCR, was notably diminished in RANKL‐induced BMMs compared to uninduced BMMs (Figure S5J–L, Supporting Information). Additionally, despite Etomoxir treatment, RANKL‐induced osteoclastogenesis was not significantly affected (Figure S5M–O, Supporting Information), potentially due to overlapping effects of FAO inhibition by Etomoxir and the natural downregulation of FAO by RANKL induction. These findings suggest that early RANKL‐induced osteoclast differentiation is characterized by activated glycolysis and suppressed FAO.

Collectively, our study demonstrates that *Prmt6* deficiency during RANKL‐induced osteoclastogenesis leads to a metabolic shift from glycolysis to FAO. This metabolic shift starkly contrasts with the profile observed in *Prmt6^+/+^* BMMs, where enhanced glycolysis and reduced FAO characterize normal osteoclastogenesis. The distinct metabolic phenotype of *Prmt6*‐deficient cells underscores the critical role of PRMT6 in mediating metabolic reprogramming between glycolysis and FAO in response to RANK signaling, ultimately influencing osteoclast differentiation and function.

### 2.6 HIF‐1α Involvement in PRMT6's Role in Promoting Glycolysis and Inhibiting FAO

Due to significant inhibition of the HIF‐1 signaling pathway and reduction in HIF‐1α levels caused by *Prmt6* deficiency, we further investigated the influence of HIF‐1α on Glycolysis and FAO under RANKL‐induced conditions. We observed that HIF‐1α overexpression partially restored the decreased expression of key glycolysis genes *Pfkm*, *Pkm*, and *Ldha* due to *Prmt6* deficiency (Figure S6A, Supporting Information), along with their protein levels (Figure S6B,C, Supporting Information). ECAR measurements at 24 h post‐induction showed a gradient increase in both basal and maximal levels in *Prmt6^−/−^* BMMs with HIF‐1α overexpression (Figure S6D,E, Supporting Information). However, HIF‐1α overexpression significantly mitigated the elevated expression of FAO‐related genes *Cpt1a*, *Acox3*, and *Acadl* observed in *Prmt6*‐deficient cells (Figure S6F, Supporting Information), as well as their protein levels (Figure S6G,H, Supporting Information). In *Prmt6^+/+^* BMMs, HIF‐1α overexpression markedly suppressed the protein levels of CPT1a, ACOX3, and ACADL, an effect reversed by the HIF‐1α inhibitor Acriflavine (Figure S6I,J, Supporting Information). OCR measurements of Etomoxir‐treated *Prmt6^−/−^* BMMs indicated that HIF‐1α overexpression significantly inhibited FAO at 24 h post‐RANKL induction (Figure S6K–M, Supporting Information). These findings suggest that HIF‐1α acts as a mediator of PRMT6's role in enhancing glycolysis and suppressing FAO during the initial stages of RANKL‐induced osteoclastogenesis.

### 2.7 PRMT6 Reprograms Metabolic Shift in RANKL‐Induced BMMs by Regulating Chromatin Accessibility

**Figure 7:**
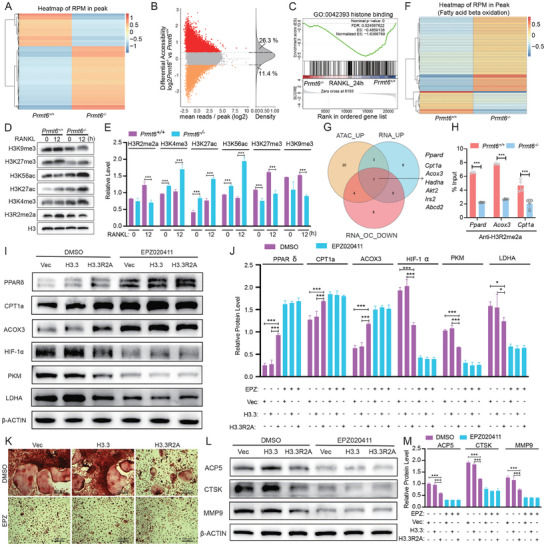
PRMT6 mediates metabolic shifts in RANKL‐induced BMMs by regulating histone epigenetic modification. A) Heatmap from ATAC‐seq analysis showing RPM across overall chromatin accessible regions (peaks) between *Prmt6^−/−^* and *Prmt6^+/+^* BMMs after RANKL induction, suggesting increased chromatin accessibility in *Prmt6^−/−^* samples. B) A differential accessibility plot illustrating the log_2_ fold change in reads per accessible region against the mean reads per region, reveals that 26.3% regions exhibit increased openness (fold change > 1.3), while 11.4% show decreased accessibility (fold change < −1.3) in RANKL‐stimulated *Prmt6^−/−^* BMMs compared to *Prmt6^+/+^* counterparts. C) GSEA of RNA‐seq data revealed altered histone binding in *Prmt6^−/−^* BMMs compared to *Prmt6^+/+^* counterparts after RANKL induction, indicating the influence of PRMT6 on histone modification. D) *Prmt6* deficiency counteracted RANKL‐induced upregulation of H3R2me2a modification, concurrently increasing active histone mark expression (H3K27ac, H3k56ac and H3K4me3) and reducing repressive marks (H3k27me3, H3K9me3) in *Prmt6^−/−^* compared to *Prmt6^+/+^* BMMs during RANKL stimulation. E) Quantitative analysis indicated that at 12 h post‐RANKL induction, repressive histone markers were significantly lower, and active histone markers were significantly higher in *Prmt6^−/−^* BMMs compared to wild‐type BMMs. F) Heatmap showing enhanced chromatin accessibility for FAO genes in *Prmt6^−/−^* compared to *Prmt6^+/+^* BMMs after RANKL stimulation, as indicated by increased RPM of associated peaks. G) Venn diagram depicting the overlap among FAO genes demonstrating both increased chromatin accessibility (ATAC‐seq) and upregulated mRNA (RNA‐seq) in *Prmt6^−/−^* BMMs relative to *Prmt6^+/+^* counterparts following RANKL induction, alongside those downregulated mRNA (RNA‐seq) in *Prmt6^+/+^* cells after RANKL treatment. H) ChIP‐qPCR analysis revealing significant enrichment of H3R2me2a modification at the promoters of key FAO genes including *Ppard*, *Cpt1a*, and *Acox3* in RANKL‐stimulated *Prmt6^+/+^* BMMs compared to *Prmt6^−/−^* cells. I,J) Western Blot (I) and quantitative analysis (J) show that overexpression of H3R2A, which blocks methylation at the H3R2 site, significantly increased the levels of FAO‐related proteins (PPARδ, CPT1a, and ACOX3) and significantly decreased the levels of glycolysis‐related proteins (HIF‐1α, PKM, and LDHA) after 24 h of RANKL induction. Under the condition of PRMT6 inhibition with EPZ020411, blocking H3R2 methylation did not significantly affect the expression levels of FAO‐related proteins, which remained relatively high, nor did it significantly affect the expression levels of glycolysis‐related proteins, which remained relatively low. K) TRAP staining indicates that overexpression of H3R2A, blocking methylation at the H3R2 site, significantly inhibited the formation of TRAP+ osteoclasts. L,M) Western Blot (L) and quantitative analysis (M) show that overexpression of H3R2A, blocking methylation at the H3R2 site, significantly inhibited the expression of osteoclast markers ACP5, CTSK, and MMP9 induced by RANKL. This effect was completely abolished by the inhibition of PRMT6 with EPZ020411.

Given PRMT6's significant role in epigenetic regulation, we aimed to determine if this mechanism influences RANKL‐induced osteoclastogenesis and metabolic reprogramming. To explore this, we examined changes in chromatin accessibility using ATAC‐seq. *Prmt6^−/−^* BMMs displayed increased chromatin accessibility at 12 h post‐RANKL stimulation compared to their *Prmt6^+/+^* counterparts (**Figure** **7**A). Specifically, 26.3% of regions were over 1.3‐fold more accessible in *Prmt6^−/−^* samples, while only 11.4% showed increased accessibility in *Prmt6^+/+^* samples (Figure 7B). Recognizing the connection between chromatin structure and histone modifications, GSEA of RNA‐seq data highlighted significant effects of *Prmt6* deficiency on histone binding (Figure 7C). Further analysis revealed that *Prmt6* deficiency negated the RANKL‐induced asymmetrical dimethylation of H3R2 and led to an increase in active histone marks (H3K27ac, H3K56ac, and H3K4me3) while decreasing repressive marks (H3K27me3, H3K9me3) (Figure 7D,E) at 12 h of RANKL induction. This indicates that PRMT6 contributes to reduced chromatin accessibility via H3R2me2a methylation.

The enhanced FAO observed in *Prmt6^−/−^* cells aligned with the increased chromatin openness resulting from *Prmt6* deficiency. This prompted us to investigate the chromatin accessibility of FAO‐related genes. Notably, *Prmt6^−/−^* BMMs exhibited greater accessibility to FAO‐related genes after 12 h of RANKL induction compared to *Prmt6^+/+^* cells (Figure 7F), suggesting that *Prmt6* deficiency promotes access to these genes. To identify specific targets of PRMT6, we conducted a Venn analysis on FAO genes that showed increased chromatin accessibility (ATAC‐seq) and upregulation (RNA‐seq) in *Prmt6^−/−^* BMMs compared to *Prmt6^+/+^* cells following RANKL induction, as well as those downregulated in *Prmt6^+/+^* cells post‐RANKL treatment (Figure 7G). This analysis identified seven genes—*Ppard*, *Cpt1a*, *Acox3*, *Hadha*, *Akt2*, *Irs2* and *Abcd2*—as potential targets of PRMT6's chromatin regulatory actions during osteoclastogenesis (Figure 7G). ChIP‐qPCR further confirmed significantly reduced H3R2me2a modification at the promoters of key FAO genes (*Ppard*, *Cpt1a*, *Acox3*) in *Prmt6^−/−^* cells, thereby facilitating increased chromatin accessibility for these genes (Figure 7H).

We next constructed a histone plasmid with an arginine‐to‐alanine mutation at arginine residues of H3.3 (H3.3R2A) to competitively inhibit methylation at the H3R2 site. Overexpression of H3.3R2A, which inhibits methylation at this site, significantly increased the expression levels of FAO‐related proteins PPARδ, CPT1a, and ACOX3 under RANKL induction. In cells treated with a PRMT6 inhibitor, inhibition of this site no longer affected the expression levels of these proteins (Figure 7I,J), suggesting that PRMT6 suppresses the expression of FAO‐related proteins through H3R2 methylation. Conversely, glycolytic proteins HIF‐1α, PKM, and LDHA were reduced following H3.3R2A overexpression, likely due to the enhanced FAO exerting an inhibitory effect on glycolysis (Figure 7I,J). Consequently, disrupting methylation at the H3R2 site interfered with PRMT6‐mediated metabolic reprogramming during RANKL‐induced osteoclast differentiation. As a result, H3.3R2A overexpression significantly inhibited the formation of TRAP+ osteoclasts (Figure 7K) and the expression of osteoclast markers ACP5, CTSK, and MMP9 (Figure 7L,M).

Collectively, these findings elucidate PRMT6's pivotal role in guiding the metabolic shift from FAO to glycolysis through epigenetic mechanisms, advancing our understanding of its function in osteoclastogenesis and metabolic reprogramming.

### 2.8 Inhibition of PRMT6 as a Therapeutic Strategy against OVX‐Induced Osteoporosis

**Figure 8:**
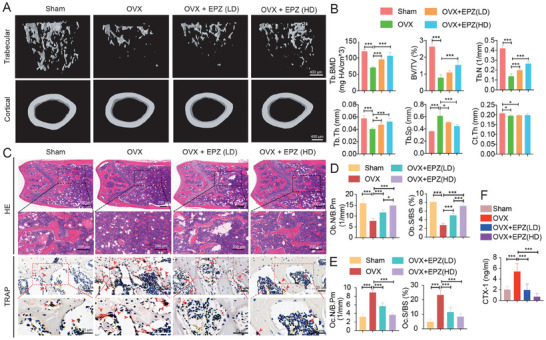
PRMT6 inhibitor (EPZ020411) treatment protects against OVX‐induced osteoporosis and osteoclastogenesis in vivo. A) Micro‐CT images of femurs from Sham, OVX, and OVX mice treated with EPZ020411 at low (LD) or high dose (HD) demonstrate that EPZ treatment significantly mitigates OVX‐induced trabecular bone loss, while its impact on cortical bone remains minimal. B) Micro‐CT derived parameters, including bone mineral density (BMD), bone volume fraction (BV/TV), trabecular number (Tb.N), trabecular thickness (Tb.Th), trabecular separation (Tb.Sp), and cortical thickness (Ct. Th) for Sham, OVX, and OVX + EPZ treated groups. C) Hematoxylin and eosin (HE) and tartrate‐resistant acid phosphatase (TRAP) stained sections of distal femurs. Upper panels: HE staining reveals the attenuation of OVX‐induced trabecular bone loss following EPZ treatment; Lower panels: TRAP staining, with red arrows highlighting TRAP+ osteoclasts, demonstrates a decrease in OVX‐induced osteoclast formation as a result of EPZ intervention. D) Quantitative analyses of osteoblast number per bone perimeter (Ob.N/B.Pm) and osteoblast surface area per bone surface area (Ob.S/BS) in distal femurs across all groups. E) Quantitative analyses of osteoclast number per bone perimeter (Oc.N/B.Pm) and osteoclast surface area per bone surface area (Oc.S/BS) in distal femurs across all groups. F) The serum level of C‐terminal telopeptide type 1 collagen (CTX‐1) was measured by ELISA assay for each group.

Given PRMT6's critical role in enhancing osteoclastogenesis both in vitro and in vivo, we investigated its potential as a therapeutic target for osteoporosis induced by OVX. Five weeks after OVX, significant uterine atrophy confirmed the success of the model (Figure S7A, Supporting Information). Importantly, treatment with the selective PRMT6 inhibitor, EPZ020411, did not affect body weight (Figure S7B, Supporting Information). Micro‐CT analysis showed that PRMT6 inhibitor treatment markedly mitigated trabecular bone loss compared to the vehicle‐treated group (**Figure** **8**A). This effect was dose‐dependent, with improvements observed in key structural parameters such as Tb.BMD, BV/TV, Tb.N, Tb.Th, and Tb.Sp (Figure 8B). However, Ct.Th remained unaffected (Figure 8B).

Histological examination further supported these findings, showing reduced trabecular bone loss and a significant decrease in the number of TRAP‐positive osteoclasts following PRMT6 inhibition (Figure 8C). Quantitative histomorphometric analysis revealed that PRMT6 inhibition alleviated the OVX‐induced reduction in Ob.N/B.Pm and Ob.S/BS (Figure 8D), while significantly lowering the OVX‐induced increase in Oc.N/B.Pm and Oc.S/BS (Figure 8E). Moreover, serum levels of the bone resorption marker CTX‐1 were reduced in the PRMT6 inhibitor‐treated group compared to the vehicle‐treated group (Figure 8F).

In summary, these results demonstrate that inhibition of PRMT6 effectively reduces OVX‐induced osteoclastogenesis and trabecular bone loss. This highlights PRMT6 as a promising therapeutic target for the treatment of osteoporosis.

## 3 Discussion

**Figure 9:**
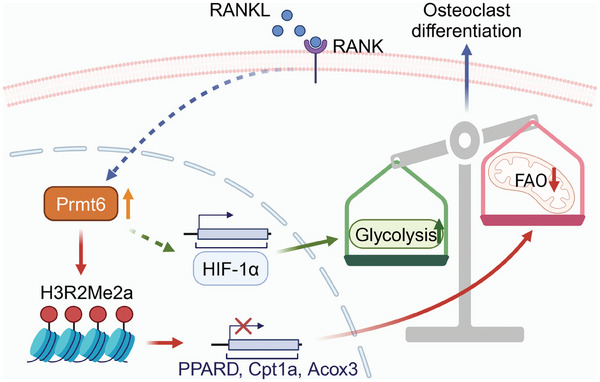
Schematic of PRMT6‐mediated epigenetic regulation in metabolic reprogramming during osteoclastogenesis. RANKL‐induced upregulation of PRMT6 leads to the asymmetric dimethylation of H3R2 at the promoters of key fatty acid oxidation (FAO) genes such as *Ppard*, *Acox3*, and *Cpt1a*. This epigenetic modification constricts genomic accessibility for FAO genes, thus inhibiting FAO activity. The reduction in FAO alleviates its suppressive effect on glycolysis, synergizing with PRMT6's promotion of HIF‐1 signaling‐dependent glycolysis to enhance cellular glycolytic activity. Consequently, a metabolic shift from FAO to glycolysis occurs, a transition that significantly contributes to osteoclast differentiation and activation.

The activation of RANK signaling plays a pivotal role in initiating osteoclastogenesis and constitutes a critical pathway implicated in various bone‐related diseases, including postmenopausal osteoporosis, periodontitis, rheumatoid arthritis, and metastatic cancers.^[^ ^33^, ^34^ ^]^ Emerging research has begun to underscore the importance of metabolic reprogramming within osteoclastogenesis, presenting a promising perspective for understanding bone resorption mechanisms.^[^ ^35^, ^36^, ^37^ ^]^ Despite these advances, the specific mechanism by which metabolic reprogramming occurs in response to RANK signaling activation remain inadequately understood. Our findings contribute to this growing and fundamental field by identifying PRMT6 as a critical metabolic checkpoint that orchestrates a metabolic shift from fatty acid oxidation to glycolysis. This process is facilitated through the modulation chromatin accessibility, thereby translating RANK signaling into a metabolic switch crucial for osteoclast differentiation (**Figure** **9**). This discovery not only enhances our understanding of the molecular underpinnings of osteoclastogenesis but also positions PRMT6 as a potential target for therapeutic intervention. The ability of PRMT6 deficiency or inhibition to impede osteoclast differentiation and mitigate bone loss in ovariectomized (OVX) mice underscores its significance as a novel target for anti‐resorptive therapy.

PRMT6 is increasingly recognized for its diverse roles in cellular identity and function, having been previously implicated in the regulation of pancreatic β cells and embryonic stem cells.^[^ ^38^, ^39^ ^]^ Our study extends the understanding of PRMT6's biological significance by demonstrating its crucial role as a promoter of osteoclast differentiation induced by RANKL. Initially, through comprehensive RNA‐seq and ATAC‐seq analyses, we observed a notable upregulation of PRMT6 in BMMs following RANK signaling activation, a finding we validated through rigorous in vitro and in vivo experiments. During this phase, *Auts2* was identified as another candidate gene, widely known for its crucial role in neurodevelopment.^[^ ^40^, ^41^, ^42^ ^]^ However, due to its significantly lower expression relative to PRMT6 in our model, we focused solely on PRMT6 for further investigation. The upregulation of PRMT6 underscores the responsiveness to osteoclastogenic stimuli, positioning it as a pivotal mediator in the process. Further investigations revealed that inhibiting or knocking out PRMT6 in BMMs significantly attenuated the formation of TRAP+ osteoclasts and the expression of key osteoclast markers upon RANKL stimulation. This effect was accompanied by a reduction in the activation of NF‐κB, p38 and ERK pathways in RANKL‐stimulated BMMs, all of which are known to play essential roles in osteoclastogenesis. These observations are in alignment with previous studies that identified PRMT6 as a co‐activator of NF‐κB, involved in the regulation of NF‐κB's nuclear translocation,^[^ ^43^, ^44^ ^]^ thereby suggesting a mechanistic link between PRMT6's regulatory functions and osteoclastogenesis. Moreover, our in vivo analyses using *Prmt6‐deficient* mice revealed a marked decrease in osteoclast formation post‐ovariectomy compared to their wild‐type counterparts. This finding not only conforms the essential role of PRMT6 in the RANK signaling pathway but also highlights its potential as a therapeutic target for conditions characterized by excessive bone resorption.

Our investigation into PRMT6's role in RANK signaling has unveiled significant insights into the metabolic reprogramming of osteoclastogenesis, particularly highlighting the HIF‐1 signaling pathway and glycolysis as key mechanistic pathways. Initial ATAC‐seq analysis revealed that among the differential peaks in *Prmt6*‐deficient versus wild‐type cells, the HIF‐1 signaling and Glycolysis/Gluconeogenesis were the most significantly enriched pathways identified within the KEGG database. This enrichment was corroborated by RNA‐seq data, which showed a pronounced downregulation of these pathways in *Prmt6^−/−^* cells. Further proteomics analysis supported these findings, indicating a diminished activation of glycolytic enzymes during osteoclastogenesis in the absence of PRMT6. The involvement of HIF‐1α, a pivotal transcription factor in the HIF‐1 signaling pathway, is particularly noteworthy. It has been well‐documented that HIF‐1α propels glycolysis by directly activating enzymes critical for this metabolic pathway, such as GLUT1, PKM2, LDHA, and PDK1.^[^ ^45^, ^46^, ^47^ ^]^ Our findings also indicated the intimate linkage between HIF‐1 signaling and glycolysis in the context of RANK signaling activation, evidenced by their simultaneous alteration in our omics analyses and partially rescued glycolysis by HIF‐1α overexpression in *Prmt6*‐deficent BMMs. Moreover, our data supported that the significantly reduced HIF‐1α and HIF‐1α‐dependent glycolysis as an important contribution for impaired osteoclastogenesis caused by *Prmt6* deficiency. This aligns with existing studies highlighting the role of hypoxia and, by extension, the HIF‐1 signaling pathway in enhancing RANKL‐induced osteoclast formation,^[^ ^48^, ^49^ ^]^ whereas hyperoxic conditions have been shown to inhibit this process.^[^ ^50^, ^51^ ^]^ Furthermore, analysis of cis‐acting expression quantitative trait loci in patients with rheumatoid arthritis revealed a correlation between higher HIF‐1α expression and increased bone erosion,^[^ ^52^ ^]^ underscoring the clinical relevance of HIF‐1 signaling in bone pathology. Taken together, PRMT6 may safeguard the activation of HIF‐1 signaling pathways and glycolysis in response to RANKL induction, supporting osteoclastogenesis.

Metabolism guides cell lineage commitment.^[^ ^53^, ^54^ ^]^ Notably, accumulated evidence supports the premise that metabolic reprogramming, particularly the shift toward glycolysis, is a pivotal response to RANKL stimulation, orchestrating the process of osteoclastogenesis.^[^ ^11^, ^37^, ^55^, ^56^ ^]^ The critical role of glycolysis in this process has been reinforced by the observation that the glycolytic inhibitor 2‐DG curtails RANKL‐induced osteoclast formation, echoing findings reported by Koichi Murata in human macrophages.^[^ ^49^ ^]^ This is further substantiated by studies showing that genetic deletion of *Glut1*, thereby reducing aerobic glycolysis, significantly hampers osteoclastogenesis.^[^ ^10^ ^]^ Conversely, fatty acid oxidation, a metabolic pathway associated with IL‐4‐meidated alternative activation of macrophages, which fosters tissue repair as opposed to the LPS‐mediated classical activation linked to inflammation,^[^ ^53^ ^]^ appears to play a secondary role in osteoclastogenesis. This assertion is supported by evidence that showing various fatty acids inhibit osteoclastogenesis,^[^ ^57^, ^58^, ^59^ ^]^ suggesting fatty acid oxidation is not the primary driver in this process.

Our current study elucidates a critical metabolic switch under RANK signaling, wherein BMMs transition from fatty acid oxidation to glycolysis. This shift is reversed by *Prmt6* deficiency, indicating a preferential reliance on glucose metabolism over fatty acids oxidation during the initial stages of osteoclast differentiation, with PRMT6 acting as a key regulator of this metabolic pivot. The identification of PRMT6 as a metabolic checkpoint highlights a nuanced understanding of the metabolic dynamics governing osteoclastogenesis and underscores the therapeutic potential of modulating this metabolic switch. Mechanistically, HIF‐1α is a crucial mediator of PRMT6‐regulated metabolic switching from FAO to glycolysis. Our results suggest that HIF‐1α, beyond being a key promoter of glycolysis in response to RANK signaling activation, also participates in the inhibition of FAO. This HIF‐1α‐mediated inhibitory effect on FAO has been similarly observed in tumors, where HIF‐1α inhibits FAO by suppressing MCAD and LCAD, thus promoting tumor progression.^[^ ^60^ ^]^ Additionally, HIF‐1α has been reported to negatively regulate oxidative metabolism, for instance, by actively promoting the expression of PDK, which inhibits pyruvate dehydrogenase (PDH) activity, thereby reducing pyruvate entry into the TCA cycle and indirectly inhibiting FAO.^[^ ^61^ ^]^ In this study, the regulatory role of PRMT6 on HIF‐1α is identified as a key mechanism mediating the metabolic switch between glycolysis and FAO during the initiation of osteoclast differentiation.

Epigenetic mechanisms are increasingly recognized for their pivotal role in cellular differentiation and metabolic reprogramming.^[^ ^62^, ^63^, ^64^ ^]^ Our study advances this understanding by demonstrating that PRMT6, through its catalytic activity leading to H3R2 methylation (H3R2me2a),^[^ ^20^, ^29^, ^39^, ^65^ ^]^ significantly influences the epigenetic landscape during osteoclastogenesis. Here, we observed a marked increase in H3R2me2a levels following RANKL induction, directly implicating PRMT6 in the epigenetic regulation of RANK signaling response. Notably, the absence of PRMT6 disrupted normal H3R2 dimethylation patterns, leading to a broad epigenetic reconfiguration characterized by decreased levels of repressive histone modifications and an increase in active histone marks. These changes were paralleled by enhanced chromatin accessibility in *Prmt6*‐deficient BMMs, as revealed through ATAC‐seq analysis, suggesting a critical role for PRMT6 in mediating chromatin compacting in response to osteoclastogenic signals. This shift toward a more open chromatin state in the absence of PRMT6, indicative of a cellular identity poised for translation, reflects a departure from the compact chromatin typically associated differentiated cell states. Furthermore, our comparative analysis revealed increased chromatin accessibility at gene loci involved in FAO in *Prmt6*‐deficient cells, a finding corroborated by GSEA results which indicated upregulated FAO pathways. Additionally, we observed a pronounced enrichment of H3R2me2a at the promoters of gene loci crucial for FAO, such as *Ppard*, *Acox3* and *Cpt1a*, in PRMT6 wild‐type cells compared to their deficient counterparts. Inhibition of H3R2 methylation significantly increased the expression of key FAO proteins PPARδ, ACOX3, and CPT1a in BMMs stimulated by RANKL, whereas simultaneous inhibition of PRMT6 abolished this effect. These findings elucidate the epigenetic mechanisms underpinning the shift toward FAO in cells lacking PRMT6. PRMT6 indirectly influences glycolytic activity through its epigenetic repression of FAO, given FAO's inherent capacity to inhibit glycolysis. Additionally, the impairment of HIF‐1 signaling‐dependent glycolysis, consequent to *Prmt6* deficiency, further shifts the cellular metabolic preference away from glycolysis.

Osteoporosis poses a considerable public health dilemma worldwide, a challenge poised to intensify with the aging global population.^[^ ^4^, ^66^ ^]^ Characterized by heightened bone turnover due to overactive osteoclasts, postmenopausal osteoporosis epitomizes the imbalance between bone resorption and bone formation, where the activity of osteoclasts surpasses that of osteoblasts, directly leading to pronounced bone loss. Despite the availability of anti‐resorptive medications such as bisphosphonates and denosumab, there remains a pressing need for more effective treatments, as existing therapies often do not meet clinic needs comprehensively.^[^ ^67^, ^68^, ^69^ ^]^ Here, our research identifies PRMT6 as a novel and effective target for anti‐resorptive intervention. We found that inhibiting PRMT6 effectively mitigates bone loss in OVX mice, underscoring the potential of PRMT6 inhibitors in osteoporosis management. Additionally, the lack of significant differences in bone mass between *Prmt6^+/+^* and *Prmt6^−/−^* mice under non‐pathological conditions indicates that PRMT6's role is predominantly pathological, activated by excessive RANK signaling. Physiological levels of RANK signaling may not induce PRMT6 upregulation. This is substantiated by our immunohistochemistry findings, which reveal markedly increased PRMT6 expression in OVX samples versus Sham samples. These findings pave the way for anti‐resorptive therapies that are not only effective but also phase‐specific, targeting the excessive osteoclast activity characterizing the disease's active stages. Importantly, our studies show that PRMT6 inhibition does not adversely affect osteogenic differentiation in vitro, as evidenced by unchanged ALP expression and bone mineral deposition following treatment, pointing to osteoclastogenesis inhibition as the primary mechanism by which PRMT6 inhibition preserves bone integrity in OVX‐induced osteoporosis. This dual approach of effective osteoclast inhibition without compromising osteoblast function underscores the therapeutic promise of PRMT6 targeting in the landscape of osteoporosis treatment.

## 4 Conclusion

In conclusion, our study unveils PRMT6 as a critical metabolic checkpoint that orchestrates the shift from fatty acid oxidation to glycolysis, thereby translating RANK signaling into a metabolic reprogramming event during osteoclast differentiation. Mechanistically, PRMT6 catalyzed asymmetric dimethylation of H3R2 at the promoters of key genes, including *Ppard*, *Acox3*, and *Cpt1a*. This epigenetic modification restricts genomic accessibility for fatty acid oxidation genes, thus inhibiting fatty acid oxidation. The reduction in fatty acid oxidation alleviates its suppressive effect on glycolysis, synergizing with PRMT6's promotion of HIF‐1 signaling‐dependent glycolysis to enhance cellular glycolytic activity. This culminates in a metabolic transition from fatty acid oxidation to glycolysis, a shift that plays a crucial role in supporting osteoclast differentiation and activation. The strategic inhibition of PRMT6 not only reduces RANKL‐induced osteoclastogenesis but also decreases bone loss in OVX mice, positioning it as a promising target for the development of innovative anti‐resorptive therapies.

## 5 Experimental Section

### Animals

The CRISPR‐Cas9 gene‐editing system was employed to generate *Prmt6*‐deficient mice. Specifically, two guide RNAs (5′‐ ACG AAT CCC AGC AGG CCC CG‐3′ and 5′‐GAG ATC GCC TAT GCA AGT TG‐3′) was first designed to target the single exon encoding PRMT6 on chromosome 3. Then, Cas9 mRNA and gRNAs above were microinjected into the fertilized eggs of C57BL/6J mice to obtain the F0 generation mice. These sequence‐validated F0 generation mice further mated with C57BL/6J mice to generate positive F1 generation heterozygous mice, which were subsequently bred to obtain viable homozygotes. Tail genomic DNA PCR was performed to identify the mice with expected genotype using the following primers: 5′‐ TTTCGCCGTCTGGTTTCA‐3′ and 5′‐GGTCAGGGATGCTCACTTTT‐3′ for wild type; 5′‐AGGCTACCCATACGTTCT‐3′ and 5′‐CCTTTCTCCCAGTTTCAT‐3′ for Prmt6 knockout (Figure S8, Supporting Information). All the mice were bred and maintained under specific‐pathogen‐free conditions. All animal experiments were performed according to the National Institute of Health Guide for the Care and Use of Laboratory Animals with approval from the Committee on Ethics of Medicine, Naval Medical University.

### Cell Culture

Mouse bone marrow cells (BMCs) were isolated from 4–6‐week‐old littermates by flushing the bone marrow of tibiae and femurs. Primary MSCs were obtained by culturing freshly‐isolated BMCs in α‐MEM (Hyclone, Logan, USA) containing 10% fetal bovine serum (Gibco, Grand Island, USA) and 1% penicillin and streptomycin (Gibco, Grand Island, USA) at 37 °C with an atmosphere of 5% CO_2_. The culture media was replaced every 3 days and the first generation of MSCs were treated with osteogenic medium supplemented with 50 µg/ml ascorbic acid, 5 mM β‐glycerophosphate, and 100 nM dexamethasone (Cyagen, San Francisco, USA) for osteogenic induction. In order to induce osteoclastogenesis in vitro, BMCs were first induced with 50 ng ml^−1^ M‐CSF (Peprotech, New Jersey, USA) for 3 days to form bone marrow‐derived monocytes/macrophages (BMMs), following stimulating with additional 100 ng ml^−1^ RANKL (Peprotech, New Jersey, USA) for 4–6 days. In this study, *Prmt6^+/+^* and *Prmt6^−/−^* BMMs were derived from BMCs isolated from the femurs and tibias of *Prmt6^+/+^* and *Prmt6^−/−^* littermate mice. The cells were cultured in the presence of 50 ng ml^−1^ M‐CSF for 3 days to obtain *Prmt6^+/+^* and *Prmt6^−/−^* BMMs for osteoclastogenic induction.

### Cell Counting Kit‐8 Assay

Ten microliters of Cell Counting Kit‐8 reagent (NCM Biotech, Suzhou, China) were added to each well of a 96‐well plate containing 100 µl medium and then incubated for additional 3 h at 37 °C with 5% CO_2_. The absorbance at 450 nm was measured using a microplate spectrophotometer (Bio‐Tek, Vermont, USA).

### Plasmid Construction and Transfection

*Prmt6* and *Hif‐1a* plasmids has been established previously.^[^ ^70^ ^]^ cDNA fragments encoding mouse H3.3‐3xFLAG and H3.3R2A‐3xFLAG were constructed by OBIO Biotechnology and were subcloned into a pcDNA3.1 vector. Plasmids were transfected into mouse BMMs using the jetPEI‐Macrophage DNA Transfection Reagent, following the manufacturer's instructions.

### Quantitative RT‐PCR

Total RNA of cultured cells was extracted using TRIzol (Invitrogen, California, USA) according to manufacturer's instruction and quantified by a NanoDrop2000 (Thermo Fisher Scientific, California, USA), followed by being reversed into cDNA using PrimeScript RT reagent Kit (Takara, Osca, Japan). qRT‐PCR was performed on a QuantStudio^TM^3 RT‐PCR System using SYBR Premix Ex Taq II (Takara, Osca, Japan) (Primers: Table S1, Supporting Information). Relative gene expression was determined by the 2^−ΔΔCt^ method.

### Western Blot

Cells were first washed with ice‐cold PBS and then lysed in RIPA buffer containing protease and phosphatase inhibitors (Epizyme, Shanghai, China). Lysates were further subjected to ultrasonic lysis and centrifugation with 14 000 rpm at 4 °C for 15 min. A small amount of the lysate was employed to detect the total protein concentration using a BCA protein assay kit (Epizyme, Shanghai,China), and most of remaining lysate was diluted in 5 × sodium dodecyl sulfate (SDS) loading buffer and heated at 95 °C for 10 min. Then, each sample with an equal amount of total protein was separated on 10% or 12.5% SDS‐polyacrylamide gels and were subsequently transferred to PVDF membranes by a wet transfer apparatus. The membranes were blocked in the protein free rapid blocking buffer (Epizyme, Shanghai,China) for 30 min at room temperature and then incubated overnight at 4 °C with primary antibodies (Antibodies: Table S2, Supporting Information). After 2‐h incubation with a goat anti‐rabbit or anti‐mouse IgG secondary antibody HRP conjugated at room temperature, the immunoreactive bands on the membranes were visualized with enhanced chemiluminescence (Epizyme, Shanghai, China) according to the manufacturer's instructions.

### Chromatin Immunoprecipitation

The ChIP assay was performed using the SimpleChIP® Enzymatic Chromatin IP Kit (CST, Boston, USA) according to the manufacturer's instructions. Briefly, cells were incubated in 1% formaldehyde for 10 min at room temperature for chromatin crosslinking. After adding glycine for 5 min to terminate the reaction, the cell nuclei were isolated by incubating in the cold buffer containing DTT and proteinase inhibitor cocktail for 10 min. Then, the chromatin was digested with Micrococcal Nuclease at 37 °C for 20 min and subsequently released through ultrasonic sonication. Chromatin fragments were confirmed by agarose gel electrophoresis to be 150–900 bp. Subsequently, ChIP was performed using primary antibodies against H3 (supplied in the kit), H3R2me2a (Abcam, Cambridge, UK) and IgG (supplied in the kit). Precipitated DNA samples were quantified with qRT‐PCR and data were presented as the percentage of input DNA.

### ALP and Alizarin Red Staining

MSCs induced for osteogenesis for 5 days were fixed with 4% polyoxymethylene for 15 min at room temperature and then were stained for alkaline phosphatase (ALP) activity by using an ALP assay kit (Solarbio, Peking, China) through an azo‐coupling method in the dark for 15 min. The activity of ALP was determined using an ALP microplate test kit (NJBI, Nanjing, China) according to the manufacturer's instructions. The cells used for alizarin red staining underwent 14 days of osteogenic induction. After fixation with 4% polyoxymethylene for 15 min, the cells were stained with 1% alizarin red solution (Sigma–Aldrich, St. Louis, USA) at 37 °C for 20 min and washed with PBS to remove non‐specific staining. Thereafter, mineralized nodules were dissolved with 10% cetylpyridinium chloride (Sigma, St. Louis, USA) for semi‐quantitative analysis using absorbance at 562 nm.

### TRAP Staining and Bone Resorption Assay

TRAP staining was performed when mature osteoclasts were successfully generated in the control group. The cells were sequentially fixed with 4% paraformaldehyde fixation for 15 min, treated with 0.1% Triton‐100 for 30 min, and incubated with TRAP staining solution (Servicebio, Peking, China) at 37 °C in the dark for 1 h before observation. For bone resorption assay, BMMs were seeded onto the surface of bovine cortical bone slices (JoyTech, Hangzhou, China) and induced to differentiate into osteoclasts with 50 ng ml^−1^ M‐CSF and 100 ng ml^−1^ RANKL. After the osteoclasts matured, they were further cultured for two additional days before carefully cleaning the surface of the slices and observing bone resorption pits using SEM (ZEISS, Oberkochen, Germany).

### Lactic Acid Content Assay

Cell culture supernatants were collected at 0, 6, 12, and 24 h after RANKL induction, and lactate concentration were measured using a Lactic acid content assay kit (Sangon Biotech, Shanghai, China) following the manufacturer's instructions.

### Seahorse

Extracellular acidification rates (ECAR) and Oxygen consumption rates (OCR) were measured using an XF‐96 Extracellular Flux Analyser (Agilent Tecnologies, California, USA). ECAR was measured at baseline and after addition of 10 mM glucose, 1 µM oligomycin and 50 mM 2‐deoxy‐D‐glucose (Sigma, St. Louis, USA). OCR was taken under basal conditions and following the addition of 1 µM oligomycin, 1 µM fluro‐carbonyl cyanide phenylhydrazone and 100 nM rotenone + 1 µM antimycin A (Sigma, St. Louis, USA). For FAO ratio calculation, BMMs were treated with 40 µM etomoxir or not for 1 h before OCR measurement.

### FAOBlue

Cells were incubated with 5 µM FAOBlue in serum free culture meida at 37 °C for 40 min. After two washes, blue fluorescence (Ex. 405 nm/Em.430–480 nm) was observed using a fluorescence microscope (LEICA, Weztlar, Germany), and intensity was quantified using Image J software (NIH, Bethesda, USA).

### ATAC‐Seq

Considering that alterations in chromatin structure precede gene transcription and protein expression, ATAC‐seq at 0 and 12 h post RANKL stimulation, was conducted along with RNA‐seq and Proteomics at 0 and 24 h following RANKL stimulation. ATAC‐seq was performed as previously reported.^[^ ^71^ ^]^ Briefly, nuclei were extracted from samples, and the nuclei pellet was resuspended in the Tn5 transposase reaction mix. The transposition reaction was incubated at 37 °C for 30 min. Equimolar Adapter1 and Adapter 2 were added after transposition, PCR was then performed to amplify the library. After the PCR reaction, libraries were purified with the AMPure beads and library quality was assessed with Qubit. The clustering of the index‐coded samples was performed on a cBot Cluster Generation System using TruSeq PECluster Kit v3‐cBot‐HS (Illumina, San Diego, USA) according to the manufactuer's instructions. After cluster generation, the library preparations were sequenced on an Illumina platform and 150 bp paired‐end reads were generated. Raw data of fastq format were first processed using fastp (version 0.20.0) to remove reads containing adapter, reads containing poly‐N and low‐quality reads from raw data. At the same time, Q20, Q30 and GC content of the clean data were calculated. Clean reads were aligned to the reference genome using BWA mem. Reads that were derived from mitochondrion DNA and chloroplast DNA, not properly paired and with PCR duplicates, were discarded for high quality (MAPQ ≥ 13). All peak calling was performed with MACS2 (version 2.1.0) and ChIPseeker was used to retrieve the nearest genes around the peak and annotate genomic region of the peak. Peak‐related genes can be confirmed by ChIPseeker, and then Gene Ontology (GO) enrichment analysis was performed to identify the function enrichment results by using the GOseq R package. KOBAS software was used to test the statistical enrichment of peak related genes in KEGG pathways. Peaks of different groups were merged using “bedtools merge”. The mean RPM of each group in the merge peak was calculated. Only peaks with fold change of RPM more than 2 were considered as differential peaks for KEGG enrichment analysis.

### RNA‐Seq

After total RNA was extracted by Trizol reagent, mRNA was enriched by Oligo(dT) beads and was fragmented into short fragments. Those mRNA was further reverse transcripted into cDNA with random primers. Then, the cDNA fragments were purified with QiaQuick PCR extraction kit, end repaired, poly(A) added, and ligated to Illumina sequencing adapters. The ligation products were size selected by agarose gel electrophoresis, PCR amplified, and sequenced using Illumina HiSeq^TM^ 2500. In order to get high quality data, raw reads were further filtered to remove reads containing adapters, reads containing more than 10% of unknown nucleotides, and reads containing more than 50% of low quality (Q‐value ≤ 20) bases, followed by removing the rRNA mapped reads using short reads alignment tool Bowtie2. The remaining reads were then mapped to reference genome by TopHat2 (version 2.0.3.12). Gene abundance were quantified by software RSEM and normalized by using FPKM (Fragments Per Kilobase of transcript per Million mapped reads) method.

### Label‐Free Quantitative LC/MS Proteomics

The cells were transferred into a 1.5 ml tube and lysed with DB lysis buffer (8 M Urea, 100 mM triethylammonium bicarbonate, PH 8.5), followed by ultrasonication on ice for 5 min. The lysate was centrifuged at 12 000 g for 5 min at 4 °C and the supernatant was supplemented with 10 mM DL‐Dithiothreitol for 1 h at 56 °C, and subsequently alkylated with sufficient iodoacetamide for 1 h at room temperature in the dark. Each protein sample was taken and the volume was made up to 100 µL with DB lysis buffer, followed by digesting with trypsin and 100 mM triethylammonium bicarbonate at 37 °C for 4 h. Then, trypsin and CaCl_2_ were added and digested overnight. Formic acid was mixed with the digested sample to adjust PH under 3 and centrifuged at 12 000 g for 5 min at room temperature. The supernatant was slowly loaded into the C18 desalting column, washed with washing buffer (0.1% formic acid, 3% acetonitrile) 3 times, then added with elution buffer (0.1% formic acid, 70% acetonitrile). The eluents of each sample were collected and lyophilized. Then, the lyophilized powder was dissolved and fractionated using a C18 column (Waters BEH C18, 4.6 × 250 mm, 5 µm) on a Rigol L3000 HPLC system. UHPLC‐MS/MS analyses were performed using a nanoElute UHPLC system (Bruker, Saarbrucken, Germany) coupled with a tims TOF pro2 mass spectrometer (Bruker, Saarbrucken, Germany). The all resulting spectra were searched against UniProt database by the search engines: MaxQuant (Bruker, Tims), which was further employed to filter the retrieval results. The protein quantitation results were statistically analyzed by *t*‐test.

### Ovariectomy

The littermates of 12‐week‐old female *Prmt6^+/+^* (n = 8) and *Prmt6^−/−^* (n = 8) mice were randomly involved in ovariectomy (n = 4) or sham (n = 4) operation. All experimental mice (n = 16) were anesthetized by isoflurane before surgical manipulation. Ovaries were exposed through small dorsal incisions and then were carefully removed after ligation of the accompanying vessels and oviducts. The exposed ovaries were replaced back into the peritoneal cavity for the sham group. After the wound was sutured, those mice were placed on an electric blanket at 37 °C until recovery from anesthesia. After 5 weeks, the mice were euthanized and blood samples were collected for serum isolation. Intact femurs were dissected and fixed in 4% paraformaldehyde for 2 days before storage in 70% ethanol. For inhibitor administration, 10‐week‐old female C57BL/6J mice (n = 20) were randomly divided into four groups: Sham (n = 5), OVX (n = 5), OVX+EPZ(LD) (n = 5), OVX+EPZ(HD) (n = 5). The mice were intraperitoneally treated with EPZ for 5 weeks at either 5 or 10 mg per mouse per day, corresponding to the OVX+EPZ(LD) and OVX+EPZ(HD) groups, respectively. Equal amounts of vehicle were injected into mice from both Sham and OVX groups.

### Micro‐CT

Micro‐CT scanning of distal femurs was performed using a µCT scanner of SkyScan 1076 (Bruker, Saarbrucken, Germany) with a resolution of 9 µm per pixel. For the analysis of trabecular microarchitectures, a total of 200 slices below the growth plate were detected and the parameters of BMD, BV/TV, Tb.N, Tb.Th, Tb.Sp were calculated. The femoral cortical thickness (Ct.Th) was measured by analyzing 100 slices of the mid‐diaphysis.

### Histology and Immunohistochemistry

The femurs were decalcified in 10% EDTA, dehydrated in a series of ethanol (70%–100%), embedded in paraffin and sliced into 5‐µm‐thick sections. These slices were then subjected to HE and TRAP staining. To perform IHC, the slices underwent antigen retrieval by immersion in sodium citrate buffer at 99 °C for 20 min, followed by incubation with primary antibody against Prmt6 (Santa Cruz, California, USA) overnight at 4 °C and then with HRP‐conjugated secondary antibody. Afterward, the slices were processed by a DAB kit (Beyotime Biotech, Shanghai, China) for color reaction.

### Enzyme‐Linked Immunosorbent Assay

The serum levels of CTX‐1 were examined using a CTX‐1 ELISA kit (CST, Boston, USA) according to the manufacturer's instructions.

### Statistical Analysis

All data were analyzed using SPSS 24.0 (IBM, Armonk, USA). Statistic difference was analyzed by unpaired two‐tailed Student's t test or by a one‐way analysis of variance (ANOVA) followed by the lest significant difference test. A *p*‐value < 0.05 was considered statistically significant.

### Ethics Approval Statement

All animal experiments were conducted according to the ethical policies and procedures approved by the Committee on Ethics of Medicine, Naval Medical University.

## Conflict of Interest

The authors declare no conflict of interest.

## Author Contributions

W.C., W.P., and Y.L. contributed equally to this work. X.L., C.H., H.Y., and W.C. designed the project. W.C., W.P., Y.L., and H.W. performed the experiments. L.W., B.Z., Z.L., L.H., H.M., and Y.L. analyzed the data. W.C., C.H., and X.L. wrote and edited the manuscript. All authors reviewed the manuscript.

## Supporting information

Supporting Information

## Data Availability

The data that support the findings of this study are available from the corresponding author upon reasonable request.
