# Supplementary material for: PRMT6 Epigenetically Drives Metabolic Switch from Fatty Acid Oxidation toward Glycolysis and Promotes Osteoclast Differentiation During Osteoporosis
Source: Adv Sci (Weinh). 2024 Aug 9;11(40):2403177. doi: 10.1002/advs.202403177 (PMC11516099; doi:10.1002/advs.202403177)
Supplement: Supplementary file 1 — Supporting Information [file ADVS-11-2403177-s001.pdf]

ADVANCED SCIENCE Open Access

Supporting Information

for *Adv. Sci.*, DOI 10.1002/advs.202403177

PRMT6 Epigenetically Drives Metabolic Switch from Fatty Acid Oxidation toward Glycolysis and Promotes Osteoclast Differentiation During Osteoporosis

Wenxiang Chu, Weilin Peng, Yingying Lu, Yishan Liu, Qisheng Li, Haibin Wang, Liang Wang, Bangke Zhang, Zhixiao Liu, Lin Han, Hongdao Ma, Haisong Yang, Chaofeng Han and Xuhua Lu**

Supplementary Figures and Figure Legends

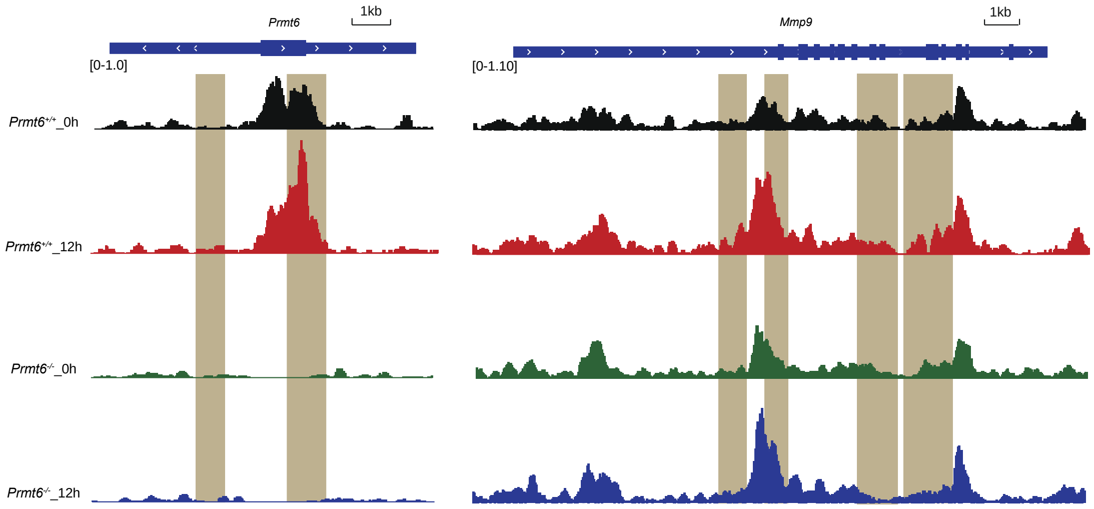

Fig. S1: Visualization of ATAC-seq normalized reads shown for *Prmt6* and osteoclast marker of *Mmp9*, with shadows highlighting changes in DNA accessibility.

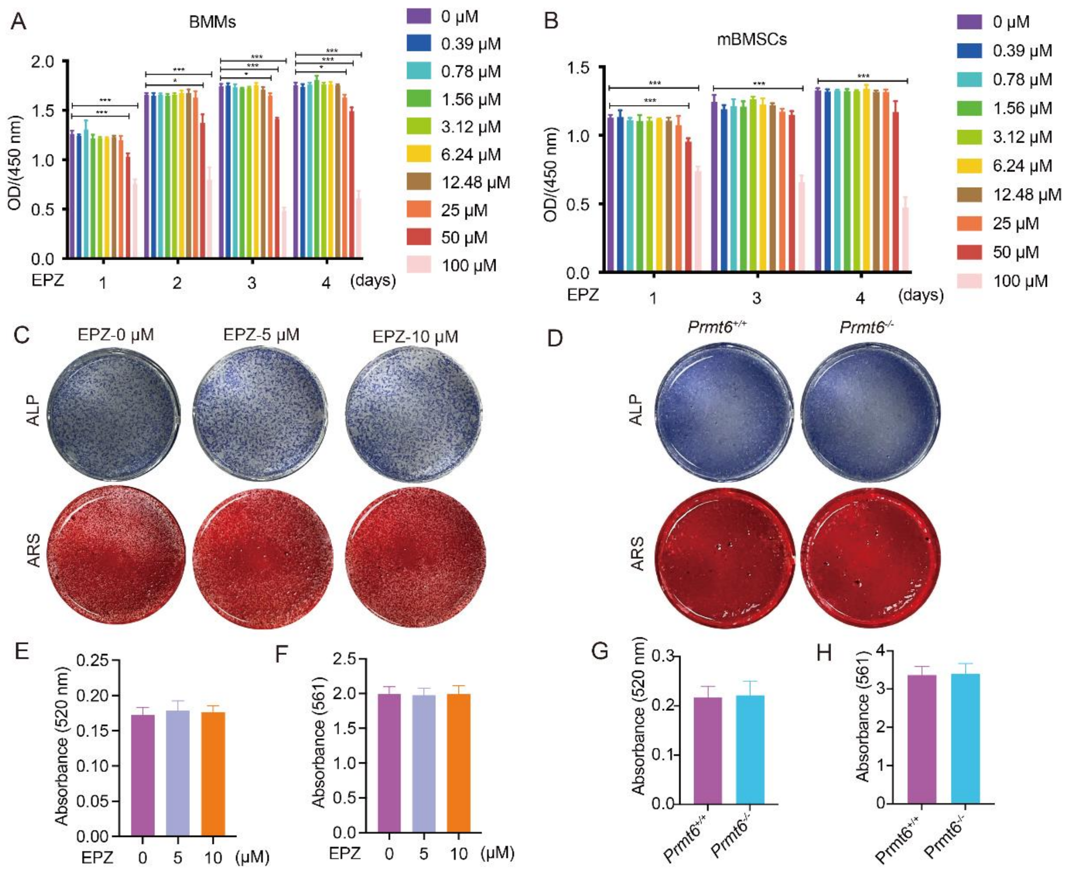

Fig. S2: Impact of PRMT6 inhibition on cell viability and osteogenesis. **A-B** Cell viability of bone marrow molecular/macrophages (BMMs) (**A**) or bone marrow mesenchymal stem cells (BMSCs) (**B**) assessed via CCK-8 assay following treatment with varying concentrations of PRMT6 inhibitor (EPZ020411). **C-D** Alkaline phosphatase (ALP) (**C**) and Alizarin Red (**D**) staining illustrate osteogenic differentiation in BMCSs treated with 0, 5, and 10 μM EPZ and in osteogenically induced *Prmt6*$^{+/+}$

versus *Prmt6*$^{-/-}$ BMSCs. **E-H** Quantitative analysis of ALP expression (**E & G**) and calcified nodule formation (**F & H**) in osteogenically induced BMSCs with EPZ treatment (**E & F**) and in *Prmt6*$^{+/+}$ versus *Prmt6*$^{-/-}$ BMSCs (**G & H**).

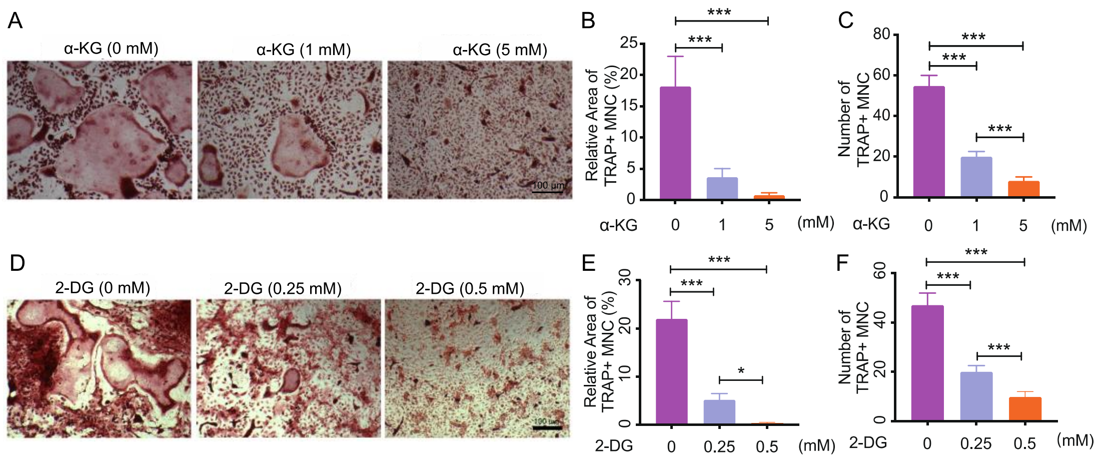

Fig. S3: HIF-1α or glycolysis inhibition impairs osteoclast formation in RANKL-induced BMMs. **A** The addition of α-KG, which depletes HIF-1α, markedly inhibited TRAP+ osteoclast formation, illustrating the crucial role of HIF-1α in osteoclastogenesis. **B-C** Quantitative analysis demonstrated the significant reduction in both the area (**B**) and number (**C**) of TRAP+ MNCs following α-KG treatment. **D** The suppressive effect of 2-DG, a glycolysis inhibitor, on tartrate-resistant acid phosphatase-positive (TRAP+) multinucleated cells

(MNs) formation. **E-F** Quantitative analysis demonstrated the significant reduction in both the area (**E**) and number (**F**) of TRAP+ MNCs following 2-DG treatment.

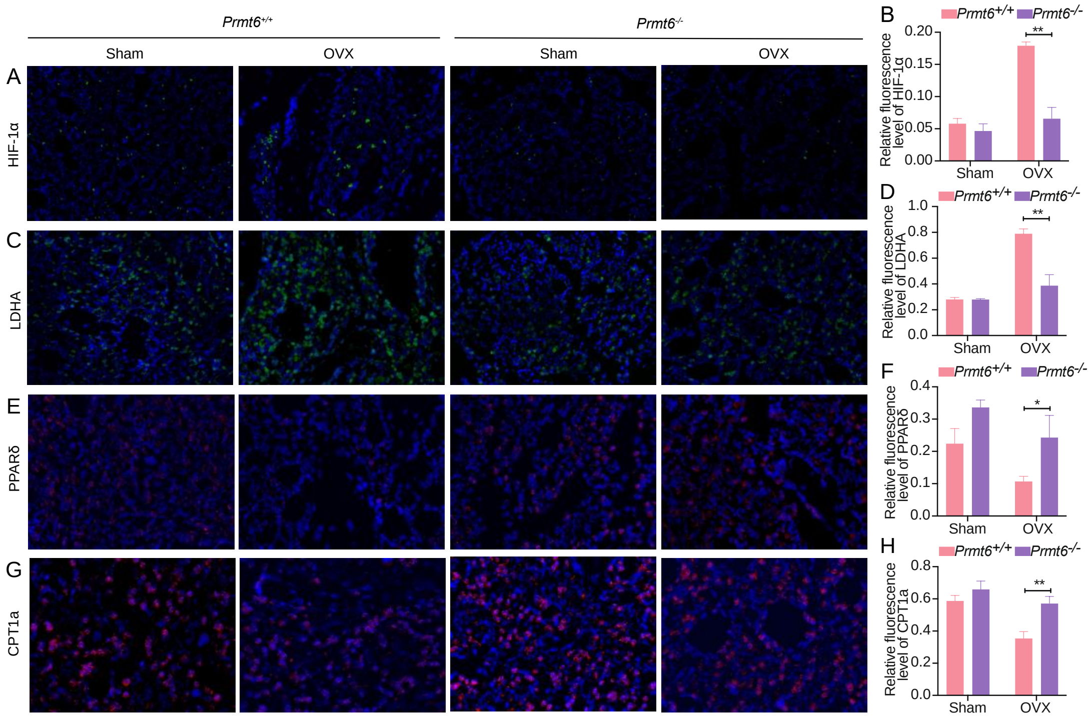

Fig. S4: *Prmt6* deficiency inhibits glycolysis activation and promotes FAO activity in vivo. **A-B** Fluorescence staining (A) and quantitative analysis (B) show that *Prmt6* deficiency significantly inhibits the overall expression levels of HIF-1α in bone marrow cells at the distal femur of OVX mice. C-D Fluorescence staining (C) and quantitative analysis (D) demonstrate that *Prmt6* deficiency significantly inhibits the overall expression levels of LDHA in bone marrow cells at the

distal femur of OVX mice. E-F Fluorescence staining (E) and quantitative analysis (F) indicate that *Prmt6* deficiency significantly increases the overall expression levels of PPARδ in bone marrow cells at the distal femur of OVX mice. G-H Fluorescence staining (G) and quantitative analysis (H) reveal that *Prmt6* deficiency significantly inhibits the overall expression levels of CPT1A in bone marrow cells at the distal femur of OVX mice.

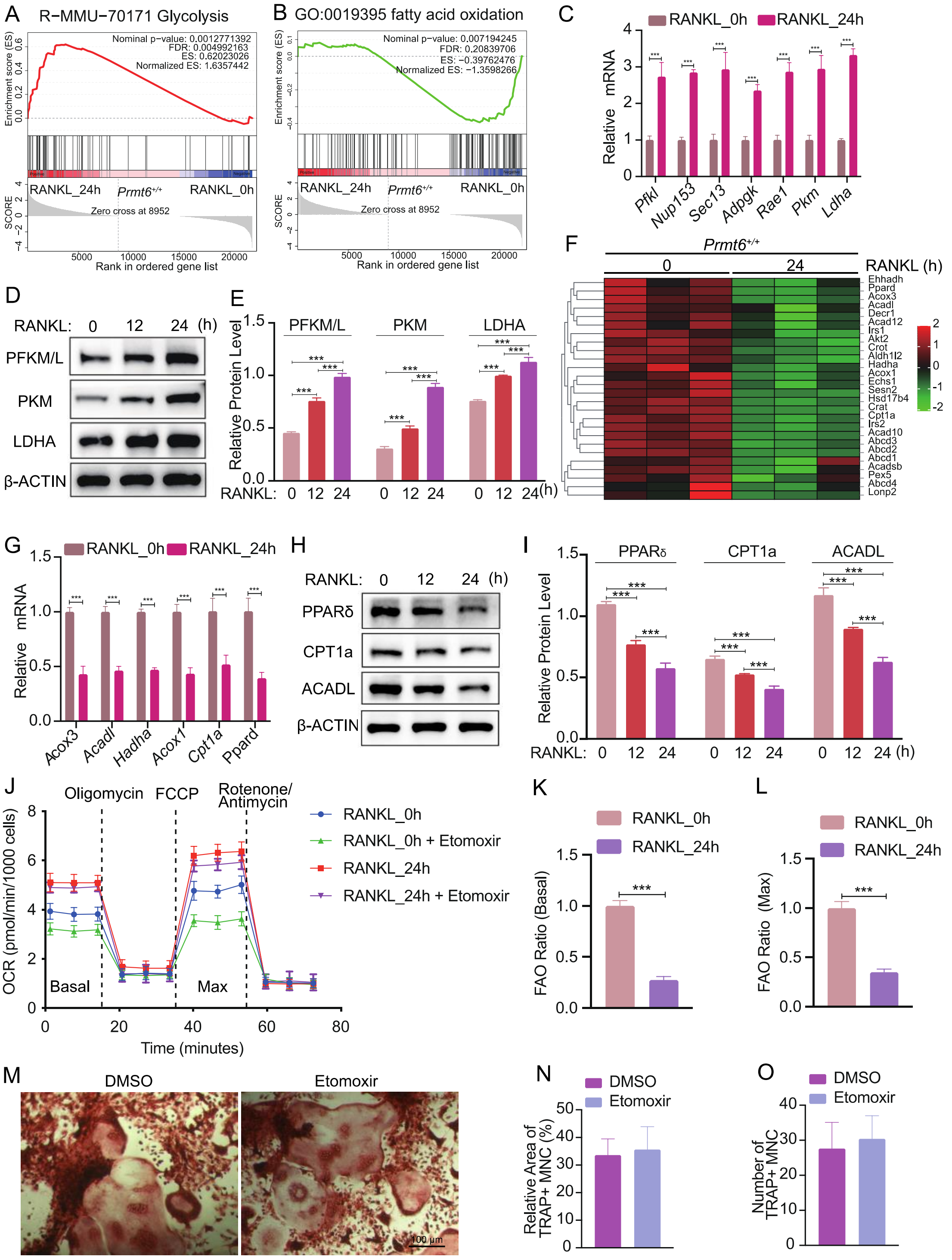

Fig. S5: Metabolic shift from FAO towards glycolysis in RANKL-induced osteogenesis at the early phase. **A-B** GSEA analysis revealed a significant upregulation of glycolysis-related genes (**A**)

and a concurrent downregulation of FAO-related genes (**B**) in *Prmt6*$^{+/+}$ BMMs 24 hours post-RANKL induction, indicating a metabolic shift from FAO towards glycolysis during RANKL-induced osteoclastogenesis. **C** qPCR analysis shows significant upregulation of glycolysis-related genes after 24 hours of RANKL induction. **D-E** Western Blot (**D**) and its quantitative analysis (**E**) indicate a significant increase in glycolysis-related proteins (PFKM/L, PKM, and LDHA) after 24 hours of RANKL induction. **F** Heatmap showcasing significantly downregulated genes involved in FAO pathway in *Prmt6*$^{+/+}$ BMMs after RANKL stimulation. **G** qPCR analysis shows significant downregulation of FAO-related genes after 24 hours of RANKL induction. **H-I** Western Blot (**H**) and its quantitative analysis (**I**) demonstrate a significant decrease in FAO-related proteins (PPARδ, CPT1a, and ACADL) after 24 hours of RANKL induction. **J** OCR quantifying FAO in BMMs before and after RANKL stimulation, with or without Etomoxir treatment. **K-L** FAO

ratio at Basal (K) and Max (L) OCR levels. **M** *Prmt6*$^{+/+}$ BMMs pretreated with 10 μM Etomoxir to inhibit FAO followed by RANKL induction showed no significant impact on osteoclast differentiation as indicated by TRAP staining. **N-O** Quantitative analysis shows that FAO inhibition does not significantly affect the relative area (**N**) and number (**O**) of TRAP+ osteoclasts.

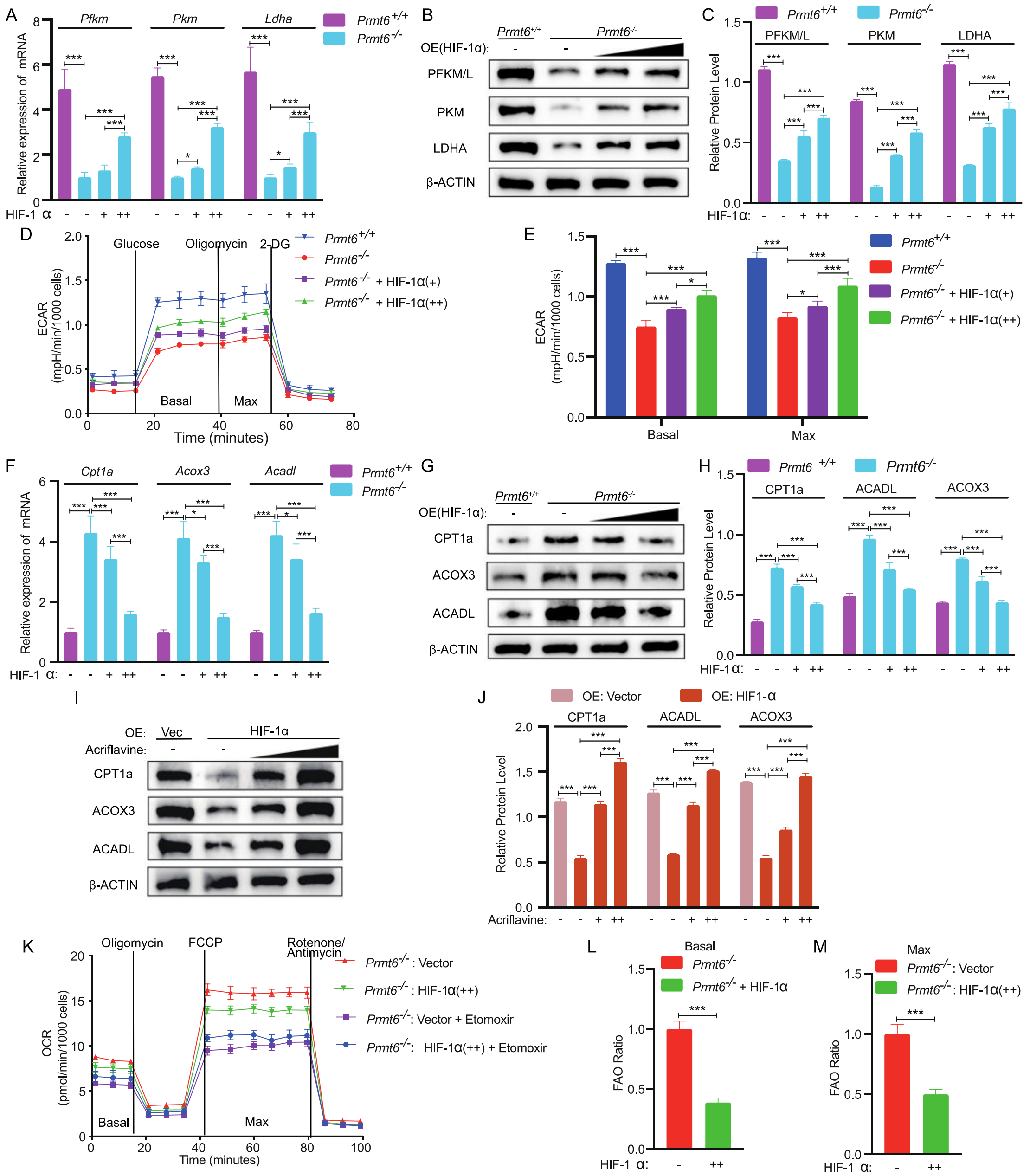

Fig. S6: HIF-1α involvement in PRMT6's role in promoting glycolysis and inhibiting FAO. **A** qPCR confirmed that HIF-1α overexpression significantly alleviated the suppression of glycolytic genes (*Pfkm*, *Pkm*, and *Ldha*) caused by *Prmt6* deficiency. **B** Western blot analysis showed that HIF-1α overexpression significantly alleviated

the suppression of glycolytic proteins (PFKM/L, PKM, and LDHA) induced by *Prmt6* deficiency. **C** Quantitative analysis indicated that HIF-1α overexpression significantly mitigated the reduction in glycolytic proteins (PFKM/L, PKM, and LDHA) caused by *Prmt6* deficiency. **D-E** Extracellular acidification rate (ECAR) measurement (**D**) and quantification (**E**) demonstrated that HIF-1α overexpression significantly rescued the ECAR reduction resulting from *Prmt6* deficiency. **F** qPCR confirmed that HIF-1α overexpression significantly inhibited the upregulation of FAO-related genes (*Cpt1a*, *Acox3*, and *Acadl*) induced by *Prmt6* deficiency. **G** WB analysis showed that HIF-1α overexpression significantly suppressed the increase in FAO proteins (CPT1a, ACOX3, and ACADL) caused by *Prmt6* deficiency. **H** Quantitative analysis confirmed that HIF-1α overexpression significantly inhibited the increase in FAO proteins (CPT1a, ACOX3, and ACADL) resulting from *Prmt6* deficiency. **I** WB analysis demonstrated that the downregulation of FAO proteins

(CPT1a, ACOX3, and ACADL) caused by HIF-1α overexpression could be reversed by the HIF-1α inhibitor Acriflavine. **J** Quantitative analysis indicated that the downregulation of FAO proteins (CPT1a, ACOX3, and ACADL) induced by HIF-1α overexpression could be reversed by Acriflavine treatment. **K** OCR quantifying FAO in 24-hour RANKL-induced Prmt6-/- BMMs overexpressing HIF-1α, with or without Etomoxir treatment. **L-M** FAO ratio at Basal (L) and Max (M) OCR levels.

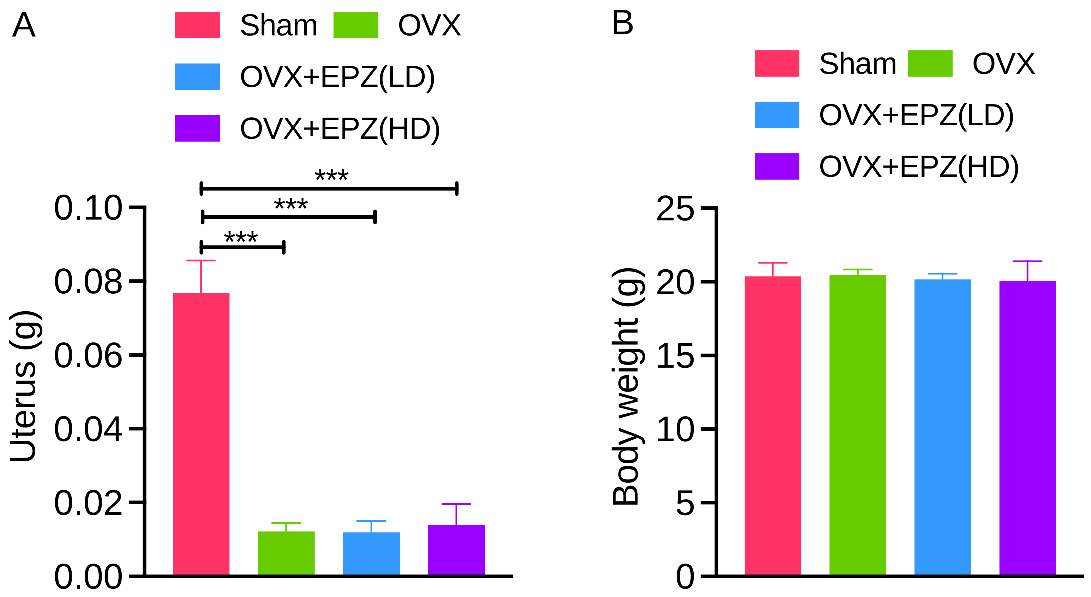

Fig. S7: Uterus weight and body weight post-PRMT6 inhibitor treatment. **A** Significant reduction in the weight of uterus observed after ovariectomy in female mice, indicating the effectiveness of the

surgical model. **B** No significant change in body weight following administration of the selective PRMT6 inhibitor (EPZ020411).

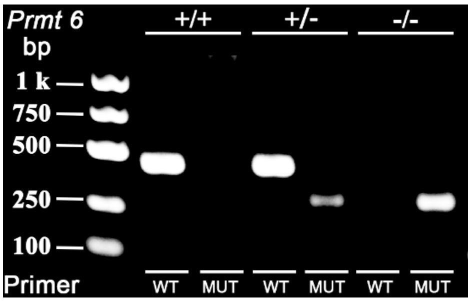

Fig. S8: Genotype identification via PCR amplification. Identification of mouse genotypes was achieved through amplification of DNA samples using separate wild type (WT) and mutant (MUT) primers. In the resulting gel electrophoresis, *Prmt6*$^{+/+}$ mice showed a distinct DNA band in the WT primer amplification lane, *Prmt6*$^{-/-}$ mice displayed a DNA band only in the MUT primer amplification lane, and *Prmt6*$^{+/-}$ mice exhibited bands in both amplification lanes.

Supplementary Tables

Table S1. Primers.

Gene	Forward (5'- 3')	Reverse (5'- 3')
Prmt6	GATGGGCTACGGACTTCTGC	GCATCTGGTCGCTAATCGGG
Ctsk	TAGCCACGCTTCCTATCCGA	CCTCCGGAGACAGAGCAAAG
Mmp9	CCCTGGAACTCACACGACAT	TGGTTCACCTCATGGTCCAC
Acp5	CACTCCCACCCTGAGATTTGT	CATCGTCTGCACGGTTCTG
Hif-1a	GATGACGGCGACATGGTTTAC	CTCACTGGGCCATTTCTGTGT
HK1	AGGGCGCATTACTCCAGAG	CCCTGTGGGTGTCTTGTGTG
Pfkl	GGAGGCGAGAACATCAAGCC	CGGCCTTCCCTCGTAGTGA
Pfkp	GAAACATGAGGCGTTCTGTGT	CCCGGCACATTGTTGGAGA
Pfkm	TGTGGTCCGAGTTGGTATCTT	GCACTTCCAATCACTGTGCC
Pkm	TGTGGTCCGAGTTGGTATCTT	GCACTTCCAATCACTGTGCC
Ldha	TGTCTCCAGCAAAGACTACTGT	GACTGTACTTGACAATGTTGGGA
Pdk1	GGACTTCGGGTCAGTGAATGC	TCCTGAGAAGATTGTCGGGGA
PPARD	TCCATCGTCAACAAAGACGGG	ACTTGGGCTCAATGATGTCAC
Cpt1a	AGATCAATCGGACCCTAGACAC	CAGCGAGTAGCGCATAGTCA
Acox3	ACCGGAAGAAAAAGACAGTGC	GAGGCTCTTGCTCGGTAGG
Abcd2	ATACACATGCTAAATGCAGCAGC	GCCAATGATGGGATAGAGGGT
Hadha	TGCATTTGCCGCAGCTTTAC	GTTGGCCCAGATTTCGTTCA
Scp2	CCTTCTGTCGCTTTGAAATCTCC	GCTTCCTTTGCCATATCAGGAT
Ivd	GGACGGCGAGTTTCCAGTT	CTCCTCGTTTAGCCCGTTGA
Acadl	TCTTTTCCTCGGAGCATGACA	GACCTCTCTACTCACTTCTCCAG
Eno1	TGCGTCCACTGGCATCTAC	CAGAGCAGGCGCAATAGTTTTA
Pgk1	ATGTCGCTTTCCAACAAGCTG	GCTCCATTGTCCAAGCAGAAT
Timp1	GCAACTCGGACCTGGTCATAA	CGGCCCGTGATGAGAAACT
Serpine1	TTCAGCCCTTGCTTGCCTC	ACACTTTTACTCCGAAGTCGGT
Cdkn1a	CCTGGTGATGTCCGACCTG	CCATGAGCGCATCGCAATC
Map2k1	AAGGTGGGGGAACTGAAGGAT	CGGATTGCGGGTTTGATCTC
Vegfa	GCACATAGAGAGAATGAGCTTCC	CTCCGCTCTGAACAAGGCT

Egln1	AGCTGGTCAGCCAGAAGAGT	GCCCTCGATCCAGGTGATCT
Camk2d	GATAAACAACAAAGCCAACGTGG	GGATTACAGTAGTTTGGGGCTC
Nup153	CGGTGAAGCCTTACCAACAGG	GTTCTCATCCGCATAAATCGCA
Sec13	GAACACTGTGGACACCTCTCA	CTCCATTCCGCACATCGAAAA
Adpgk	TCCTGGGATGGTGTTCCTGAT	GATCCTGGTGAGATCCGAGG
Rae1	TTTGGGAGCACAACCACAGAT	TAAAGTTGGCGGGCTGAAAGA
Gapdh	ACCCAGAAGACTGTGGATGG	CACATTGGGGGTAGGAACAC
Actin	ACAGCAGTTGGTTGGAGCAA	ACGCGACCATCCTCCTCTTA

Table S2. Antibodies.

Antibodies	Source	Country	Identifier
Anti-Prmt6	CST	USA	Cat# 14641
Anti-Prmt6	Santa Cruz	USA	Cat# sc-271744
Anti-GAPDH	CST	USA	Cat# 5174
Anti-Ctsk	Abclonal	China	Cat# a1782
Anti-Acp5	Abclonal	China	Cat# a2528
Anti-Mmp9	Abclonal	China	Cat# a2095
Anti-IkB	CST	USA	Cat# 4814
Anti-p-p65	CST	USA	Cat# 3033
Anti-p65	CST	USA	Cat# 8242
Anti-Erk	CST	USA	Cat# 4696
Anti-p-Erk	CST	USA	Cat# 8544
Anti-P38	CST	USA	Cat# 8690
Anti-p-P38	CST	USA	Cat# 9216
Anti- Jnk	CST	USA	Cat# 9252
Anti- p-Jnk	CST	USA	Cat# 4668
Anti-Hif-1α	Abcam	UK	Cat# ab179483
Anti-HK1	CST	USA	Cat# 2024
Anti-PFKM/L	Abcam	UK	Cat# ab181064
Anti-PKM	Abcam	UK	Cat# ab150377
Anti-LDHA	Abcam	UK	Cat# ab52488

Anti-β-Actin	CST	USA	Cat# 3700
Anti-H3	CST	USA	Cat# 4499
Anti-H3R2me2a	Abcam	UK	Cat# ab175007
Anti-H3K4me3	CST	USA	Cat# 9751
Anti-H3K27Ac	CST	USA	Cat# 8173
Anti-H3K56Ac	CST	USA	Cat# 4243
Anti-H3K9me3	CST	USA	Cat# 13969
Anti-H3K27me3	CST	USA	Cat# 9733
Anti-PPARδ	Abcam	UK	Cat#ab8937
Anti-CPT1a	Abcam	UK	Cat#ab128568
Anti-ACOX3	Invitrogen	USA	Cat#PA5-22373
Anti-ACADL	Abcam	UK	Cat#ab128566
